# On the rank-distance median of 3 permutations

**DOI:** 10.1186/s12859-018-2131-4

**Published:** 2018-05-08

**Authors:** Leonid Chindelevitch, João Paulo Pereira Zanetti, João Meidanis

**Affiliations:** 1 Simon Fraser University, Burnaby, Canada; 2 University of Campinas, Campinas, Brazil

**Keywords:** Genome rearrangements, Median problem, Polynomial-time solvability

## Abstract

**Background:**

Recently, Pereira Zanetti, Biller and Meidanis have proposed a new definition of a rearrangement distance between genomes. In this formulation, each genome is represented as a matrix, and the distance *d* is the *rank distance* between these matrices. Although defined in terms of matrices, the rank distance is equal to the minimum total weight of a series of weighted operations that leads from one genome to the other, including inversions, translocations, transpositions, and others. The computational complexity of the median-of-three problem according to this distance is currently unknown. The genome matrices are a special kind of permutation matrices, which we study in this paper.

In their paper, the authors provide an $O\left (n^{3}\right)$ algorithm for determining three candidate medians, prove the tight approximation ratio $\frac {4}{3}$, and provide a sufficient condition for their candidates to be true medians. They also conduct some experiments that suggest that their method is accurate on simulated and real data.

**Results:**

In this paper, we extend their results and provide the following:

- Three invariants characterizing the problem of finding the median of 3 matrices
- A sufficient condition for uniqueness of medians that can be checked in *O*(*n*)
- A faster, $O\left (n^{2}\right)$ algorithm for determining the median under this condition
- A new heuristic algorithm for this problem based on compressed sensing
- A $O\left (n^{4}\right)$ algorithm that exactly solves the problem when the inputs are orthogonal matrices, a class that includes both permutations and genomes as special cases.

**Conclusions:**

Our work provides the first proof that, with respect to the rank distance, the problem of finding the median of 3 genomes, as well as the median of 3 permutations, is exactly solvable in polynomial time, a result which should be contrasted with its NP-hardness for the DCJ (double cut-and-join) distance and most other families of genome rearrangement operations. This result, backed by our experimental tests, indicates that the rank distance is a viable alternative to the DCJ distance widely used in genome comparisons.

**Electronic supplementary material:**

The online version of this article (10.1186/s12859-018-2131-4) contains supplementary material, which is available to authorized users.

## Background

The present paper advances the study of genome evolution by rearrangements. Genomes are a collection of linear chromosomes (e.g., in human), circular chromosomes (e.g., in *E. coli*), or a combination of linear and circular chromosomes (e.g., in *Borrelia burgdorferi*, the etiological agent of Lyme disease [1]). Higher organisms such as eukaryotes tend to have linear chromosomes, while circular chromosomes are found in bacteria and other prokaryotes. Cell organelles also have circular chromosomes, even in higher organisms. The techniques we use in this paper apply equally well to all cases, regardless of chromosome type, since we focus on the adjacencies between consecutive syntenic regions.

Evolution can be thought of as a two-part process: (1) the natural variability of genomic processes leads to the occurrence of mutations within a population, and (2) one or more of these mutations are *selected* by the environment, meaning that the individuals carrying them survive to leave descendants. The mutations observed in genome evolution include point mutations, inversions, translocations, transpositions, duplications, horizontal gene transfer, and gene gain and loss, to name the most common ones. The next section elaborates on some of these operations.

Our focus here will be on events that *preserve the gene content*. We plan to study variations of the methods proposed here to address the important issue of gene content-changing mutations in the future. For the purposes of this paper, we do not consider gene content-changing events such as duplications, gene gain and loss, etc.

We also focus on *rearrangements*, or *large-scale changes*, that is, events that affect the position or the orientation of large, continuous regions in the genome. This excludes point mutations and small insertions and deletions, for instance. Although point mutations are important evolutionary events, in some cases genomes evolve more rapidly in structure than in sequence, resulting in a more reliable evolutionary assessment when we focus on rearrangements [2].

We briefly summarize previous work on the bioinformatics of genome rearrangements to date. A more complete account can be found in the survey by Moret, Lin, and Tang [3]. The starting point was the realization that genes are arranged in chromosomes in a linear fashion [4]. From this observation, scientists began to estimate genetic distances between genes and other markers by means of recombination (crossing-over) frequencies, a practice that continues to this day. Soon, the possibility of reconstructing evolutionary history from inversions was noticed [5]. With the advent of large-scale DNA sequencing, scientists had a richer body of data on which to base their research.

At first, it seemed hard to tackle the whole set of possible rearrangements, so only one or two important events (or operations) were considered, e.g., inversions [6–8]. However, over time, ways of taking into account many different operations were introduced [9], sometimes with weights assigned to them to roughly reflect their relative frequency. Our research is on a distance measure that tries to capture all genome rearrangement events that maintain gene content.

### Rearrangement operations

Inversions seem to be the single most common rearrangement operation that has been observed in nature. We briefly cite three examples to illustrate this point: one with plant chloroplasts, another with the mammalian X chromosome, and one with bacterial laboratory strains. Palmer and Herbon have shown that 3 inversions are enough to explain the differences between chloroplast genomes of *Brassica oleraceae* and *Brassica campestris* [2]. Back in 1988, large-scale DNA sequencing was still expensive, and their analysis was all done with restriction site mappings. They went on to determine the most parsimonious number of rearrangements separating other *Brassica* chloroplast genomes, and constructed a phylogenetic tree with 7 species. Pevzner and Tesler show an optimal rearrangement scenario involving 11 long syntenic regions between human and mouse X chromosomes, with 7 inversions [10]. Inversions have even been observed in laboratory strains. The strain K12 W3110 of *Escherichia coli*, created in 1956 in Barbara Bachmann’s lab [11], was found to contain a large inversion relative to its parent, involving roughly 20% of its genome, 24 years later, in 1981 [12].

Transpositions are also significant, but here we will only be concerned with the kind that moves blocks, rather than copies blocks, since the latter would imply a modification of the gene content. Translocations, both reciprocal and nonreciprocal, can also be selected for by the environment and go to fixation in a lineage. They produce changes in linkage and may disrupt coding regions, and have been associated with several human conditions, including Down’s syndrome, which in 5% of the cases is related to a type of translocation called the *Robertsonian translocation* [13].

Translocations can mediate chromosome fusion [13]. When two chromosomes with very small arms are translocated, sometimes the result is a larger chromosome next to a very small chromosome, which may be lost. This is thought to be a major reason for different chromosome numbers across a wide range of taxa, including primates. Human chromosome 2 is in fact homologous to a pair of chromosomes in chimpanzee, gorilla, and orangutan [14]. The pair was probably fused in the lineage leading to human.

Chromosome fission is also thought to have occurred in primate evolution. Human chromosomes 14 and 15 probably separated from a single chromosome in an ancestor 10 to 25 million years ago [15]. The macaque (*Macaca mulatta*) chromosome 7 mostly resembles this ancestral chromosome [16].

Although we have no knowledge of chromosome linearization or circularization in nature, both have been successfully achieved in the laboratory. Volff et al. managed to construct a circular-chromosome version of *Streptomyces lividans*, which normally has a linear chromosome [17]. Cui et al., on the other hand, report on an *Escherichia coli* strain with a linear genome [18].

### Modeling rearrangements

**Fig. 1:**
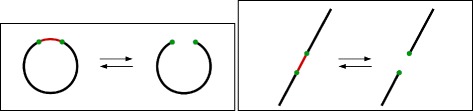
The simplest form of rearrangement mutation: adjacency creation or destruction. Left: linearization or circularization of chromosomes. Right: linear chromosome fission or fusion. The red adjacency is acquired or lost in both cases

All rearrangement events discussed so far can be seen as creating, destroying, or replacing *adjacencies*, which are links between genome segments. The simplest operation from the mutational point of view (that is, the one that changes less adjacencies) is the creation or destruction of a single adjacency (see Fig. 1). It models circularization, linearization, chromosome fusion, and chromosome fission.

**Fig. 2:**
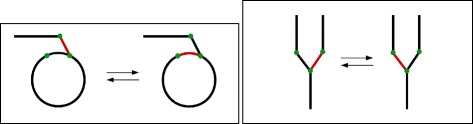
Replacement of an adjacency by another one sharing an extremity. Left: integration or excision of a circular piece at the end of a linear chromosome. Right: Nonreciprocal translocation. The red adjacency is exchanged for the black adjacency between green dots in both cases

Next in modification complexity is the replacement of a single adjacency by another one sharing an extremity (see Fig. 2). When involving linear chromosomes, it can be seen as a case of nonreciprocal translocation. An analogous case involving a circular chromosome and a linear chromosome exists, and can be viewed as circular DNA integration or excision at the very end of a linear chromosome.

**Fig. 3:**
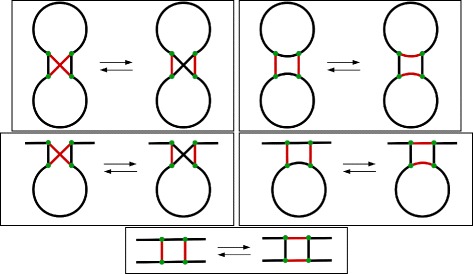
Replacement of two adjacencies by two other adjacencies sharing the same four extremities. From top to bottom, and left to right on each row: inversion on a circular chromosome; integration/excision on a circular chromosome; inversion on a linear chromosome; integration/excision on a linear chromosome; reciprocal translocation. The red adjacencies are exchanged for the black adjacencies between green dots in all cases

Finally, there are operations consisting in the replacement of two adjacencies by two other adjacencies involving the same four extremities (see Fig. 3). This class of operations include inversions (both on linear and circular chromosomes), reciprocal translocations, and other, more exotic operations that resemble the integration or excision of circular DNA into a linear or circular chromosome, except that in nature this occurs with relatively small pieces of circular DNA (e.g., plasmids, phages, and viruses in general), whereas here we are postulating that this occurs with circular DNA of any size. Such an operation has been called a *2-break* [19], a *double swap* [20], or a *double-cut-and-join* (DCJ), thus giving rise to the DCJ distance [9].

In summary, we end up with a set of operations that model a significant portion of the rearrangements that occur in nature, but also model some rearrangements that are not seen often, or that seem awkward. Although scientists are always working on providing more realistic sets of operations, a balance has to be reached between modeling power and the ability to design efficient algorithms, and this is the state-of-the-art concerning current research in the bioinformatics of genome rearrangements.

## Genomic distances

A number of genomic distances have been defined that consider the operations described in the last section. These distances differ solely by the *weight* they assign to each class of operations. Although defined in very different ways, Each such distance can be shown to be equal to the *minimum total weight* of a series of operations that corresponds to the differences seen in two genomes *A* and *B*. This suggests that these distances may be good indicators of the amount of evolution that separates the two genomes, as measured by the weight of a minimal series of events transforming *A* into *B*.

For the purposes of this paper, we focus on just four of these distances: the DCJ distance [9], the algebraic distance [21], the SCJ distance [22], and the rank distance [23]. Table 1 shows the weights each of these distances associates with each class of events.

**Table 1:** Basic rearrangement operations and corresponding weights assigned to them by the DCJ, algebraic, SCJ, and rank distances

	Distances			
Operation	DCJ	Algebraic	SCJ	Rank
Adjacency creation/destruction	1	0.5	1	1
Single adjacency replacement	1	1	2	2
Double adjacency replacement	1	1	4	2

The first observation is that the algebraic distance and the rank distance differ only by a scalar factor:

$$d_{rank}(A, B) = 2 d_{alg}(A, B). $$

This is intuitively clear from Table 1, but a formal proof can be found in the literature [23]. The meaning of this formula is that, although the two distances have been defined in very different ways, they are equivalent for all practical purposes. Everything we say about the rank distance translates in a very straightforward way to the algebraic distance, and vice-versa. For instance, solving the genome median problem (the main topic of this paper) for the algebraic distance is equivalent to solving it for the rank distance, and so on.

**Fig. 4:**
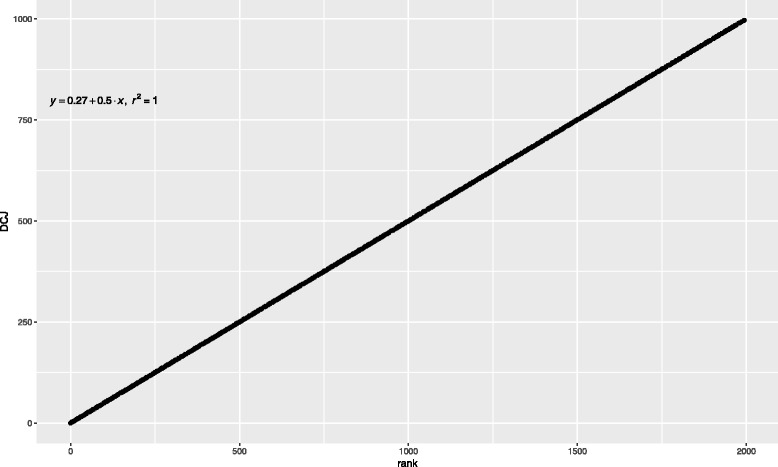
Scatterplot of the DCJ and rank distance between a “standard” and a randomly generated genome of sizes from 2 to 1000 genes. The equation of the least-squares line of best fit is also shown. Note that the slope is indistinguishable from $\frac {1}{2}$ and *r*^2^ is indistinguishable from 1

Regarding the DCJ and algebraic distances, we notice that they differ only in the weight given to adjacency creation or destruction. In practice, this turns out to be a very small difference in general. To begin with, they are equal for circular genomes (genomes containing circular chromosomes only). A scatterplot of DCJ versus rank distance for random linear genomes of various sizes, from 2 to 1000 genes (see Fig. 4), shows that they correlate extremely well for linear genomes too.

In more technical terms, the difference of 0.5 in adjacency creation or destruction has more interesting consequences. One of them is that, while computing medians under DCJ is NP-hard [24], we show in this paper that we can compute rank medians in polynomial time. It is true that the resulting median, in the case of rank distance, is not always genomic, but it is always an orthogonal matrix, and this leads to an interpretation of these medians as probability distributions over adjacencies to be chosen for a genomic near-median (see “Results” section). It is also true that the NP-hardness of the median problem for DCJ has not prevented its successful use in practice, where we see clever algorithms that can compute DCJ medians and propose ancestral genomes in a given tree (which is one of the foremost applications of medians) running in a matter of minutes to a few hours for genomes with thousands of syntenic regions [25]. However, due to the fact that the rank definition uses matrices, which are widely used and therefore have a lot of code written to deal with them, we were able to specify the rank median algorithm in just 6 lines (see Algorithm 1 below), as opposed to the complex code needed to compute DCJ medians. In fact, we believe that the adequate subgraph techniques used in fast implementations of DCJ median solvers can also be successfully applied to rank median computations.

**Fig. 5:**
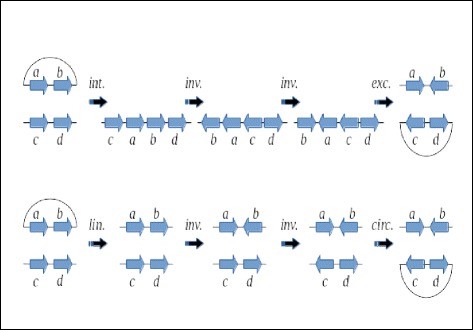
The DCJ distance does not care about mixing components, but the rank distance never mixes them. Here we see two series of operations transforming a fictitious genome *A* into a fictitious genome *B*. Both genomes have two chromosomes, one containing genes *a* and *b*, and the other containing genes *c* and *d*. (Top) This series of operations involves an integration, two inversions, and an excision of a circular piece. Notice how it mixes the two chromosomes right after the first operation. The total DCJ score of this series is 4, which is optimal for DCJ. However, the total rank score for this series is 8, which is not optimal for rank. (Bottom) This series of operations involves a linearization, two inversions, and a circularization. Notice that this process actually mutates each chromosome independently, without mixing them. Its total DCJ score is 4, which is optimal for DCJ. Its rank score is 6, which is optimal for rank. Therefore, as far as DCJ is concerned these two scenarios are equivalent, but for the rank distance only the bottom one is optimal

Another noteworthy consequence is that the rank distance never recombines graph components, while the DCJ distance sometimes does [26]. For instance, consider the example depicted in Fig. 5. Here we have two fictitious genomes, *A* and *B*, and two distinct series of operations transforming *A* into *B*. Both genomes have two chromosomes, one containing genes *a* and *b*, and the other containing genes *c* and *d*. It would seem logical to expect that, since the chromosomes do not have any genes in common, each chromosome would be treated independently of the other. Because the rank distance favors linearizations and circularizations (weight 1) over integration or excision of circular pieces of DNA (weight 2), it ends up favoring operations that deal with a single chromosome rather that mixing chromosomes, when there is a choice. In the case of Fig. 5, the rank distance favors the transformation series at the bottom, which does not mix chromosomes, while for the DCJ distance both transformation series are equally good.

But perhaps a deeper consequence is that the matrix formulation of genomes used for the rank distance provides a framework in which it is easier to draw conclusions and get insights. For instance, we prove here that if a genome *B* lies on a shortest path between genomes *A* and *C* with respect to the rank distance, then we can write

1 $$  B = A + P(C-A),  $$

for a certain matrix *P*. This resembles a similar equation that describes the line segment between two points in a multi-dimensional space:

$$\mathbf{m} = \mathbf{x} + \mu (\mathbf{y} - \mathbf{x}), $$

and can be used, for instance, to show that if an adjacency *xy* is present in both *A* and *C*, then it must be present in *B* (see Lemma 1).

## Theoretical bounds for the ratios between distances

As we saw, the rank distance is closely related to other distances that have traditionally been used in genome studies. Using multi-genome breakpoint graphs, we can derive formulas for the DCJ, algebraic, SCJ, and rank distances based on graph elements, as follows. From these formulas, we can derive theoretical bounds for the ratios between the different distances.

The multi-genome breakpoint graph is a graphical way of representing one or more genomes, but it is mainly used for two genomes *A* and *B*. The graph has a vertex for each syntenic region endpoint, and the edges correspond to the genomes’ adjacencies, using a different color for each genome. If we draw just one genome, we have a *matching*; conversely, each matching defines a unique genome. If we draw two genomes, since each one is a matching, we end up with a collection of paths and cycles. We can draw any number of genomes in this way. This is analogous to the breakpoint graph traditionally used to study genome rearrangements [8]. Note, however, that our multi-genome breakpoint graphs do not contain caps to close linear chromosomes. The ends of linear chromosomes are just extremities without adjacencies.

The SCJ distance is defined as the number of adjacencies that belong to exactly one of the two genomes. Therefore, we can compute the SCJ distance by counting all adjacencies (edges) in the multi-genome breakpoint graph and subtracting the number of common adjacencies. A common adjacency will appear in the graph as a 2-cycle, that is, a cycle composed of two parallel edges. The formula for the SCJ distance is then:

$$ d_{SCJ}(A, B) = m - 2c_{2}, $$

where *m* is the total number of edges (*A*’s plus *B*’s) and *c*_2_ is the number of 2-cycles in the graph.

The multi-genome breakpoint graph for *A* and *B* is a collection of paths and cycles. It can be shown that each path contributes its number of edges to the rank distance, and each cycle contributes its number of edges minus 2 to the rank distance. Therefore, the rank distance is:

$$ d_{rank}(A, B) = m - 2c, $$

where *c* is the number of cycles of any length in the graph. From these equations it is easy to derive the following relationship between the SCJ and rank distances:

$$ d_{rank}(A, B) \leq d_{SCJ}(A, B) \leq 2_{rank}d(A, B). $$

These inequalities are tight, as witnessed by cases in which the graph has no cycles (for the leftmost inequality), and graphs composed solely of 4-cycles (for the rightmost inequality).

With respect to the DCJ distance, it can be shown that

$$ d_{DCJ}(A, B) = \frac{1}{2}m - c + \frac{1}{2}p_{odd}, $$

where *p*_*odd*_ is the number of paths of odd length (number of edges) in the graph. From these equations it is easy to derive the following relationship between the DCJ and rank distances:

$$ d_{rank}(A, B) \leq 2d_{DCJ}(A, B) \leq 2d_{rank}(A, B). $$

These inequalities are tight as well, as witnessed by graphs with no paths (leftmost inequality), and by graphs composed solely of paths of length 1 (rightmost inequality).

Notice, however, that these theoretical bounds are very wide, typically stating that a certain distance is between 1 and 2 times some other distance, without any indication on what their actual relationship is for, say, close genomes, or genomes drawn at random. Therefore, these equations are mainly of theoretical interest and should be used with caution. We mention them here for the sake of completeness.

## The genome median problem

The genome median problem asks, given as input three genomes *G*_1_,*G*_2_,*G*_3_ and a rearrangement distance (metric) *d*, to find the *median* genome *G* minimizing $\sum _{i=1}^{3} d(G, G_{i})$. This problem has been extensively studied due to its many applications in phylogenetics and ancestral genome reconstruction [27–29], and is NP-hard for all but a few known distances *d* [22, 24, 30, 31]. Many approximation algorithms have been developed for this problem [30, 32], and some heuristic approaches have also been successfully applied to this problem [29].

Recently, Pereira Zanetti, Biller and Meidanis [23] have proposed a new definition of a rearrangement distance. In this formulation, each genome is represented as a permutation on *n* elements that is the product of disjoint cycles of length 1 (*telomeres*) and length 2 (*adjacencies*). The permutations are converted into their matrix representation, and the distance *d* is the *rank distance* between these matrices.

In their paper, the authors provide an $O\left (n^{3}\right)$ algorithm for determining three candidate medians, prove the tight approximation ratio $\frac {4}{3}$, and provide a sufficient condition for their candidates to be true medians. They also conduct some experiments that suggest that their method is accurate on simulated data.

In this paper, we extend their results and provide the following:

- Three invariants characterizing the problem of finding the median of 3 matrices
- A sufficient condition for uniqueness of medians that can be checked in *O*(*n*)
- A faster, $O\left (n^{2}\right)$ algorithm for determining the median under this condition
- A new heuristic algorithm for this problem based on compressed sensing
- an $O\left (n^{4}\right)$ that exactly solves the problem when the inputs are orthogonal matrices, a class that includes genomes as a special case

Our work thus settles the main problem of determining the complexity of the median of 3 genomic matrices, or more generally permutation matrices, with respect to the rank distance. In general, the medians that our algorithms identify are not guaranteed to be genomic matrices; however, we demonstrate that empirically, they frequently are, and when they are not, they can be converted back to genomes with minimal loss with respect to the total distance.

### Definitions and invariants

We are mostly interested in working over $\mathbb {R}$, the field of real numbers, although our results remain valid for any characteristic 0 field. On the other hand, although the problem can also be posed in finite fields, the existence of self-orthogonal vectors in these fields is likely to invalidate most of our constructions.

Let $n \in \mathbb {N}$ be an integer and let $\mathbb {R}^{n \times n}$ be the set of *n*×*n* matrices with entries in $\mathbb {R}$.

### Special matrices

We consider some special classes of matrices. We say that a matrix $M \in \mathbb {R}^{n \times n}$ is

- *Symmetric* if *M*^*T*^=*M* (i.e. *M*_*ij*_=*M*_*ji*_ ∀ *i,j*)
- *Orthogonal* if *M*^*T*^=*M*^−1^ (i.e. the columns of *M* are pairwise orthogonal)
- *Binary* if *M*_*ij*_∈{0,1} ∀ *i,j*

A matrix that is both binary and orthogonal must have a single 1 in each column and each row; it is therefore a *permutation* matrix. It defines a permutation *π* via the relationship

$$\pi(i) = j \iff M_{i,j} = 1. $$

Lastly, following [23], we say that a matrix *M* is *genomic* if it is a symmetric permutation matrix. Note that, unlike [23], we do not require *n* to be even for *M* to be genomic.

It is easy to see that by symmetry, the permutation *π* corresponding to a genomic matrix is also its own inverse, so it must have order 2 and hence is a product of disjoint cycles of length 1 and 2. The cycles of length 1 correspond to *telomeres* while the cycles of length 2 correspond to *adjacencies*. The correspondence between a genome *G* and a genomic matrix *M* is defined by

$$\begin{aligned} M_{i,j} &= 1 \; \iff \; (i,j) \; \text{is an adjacency in} \; G, \; \text{or} \\ i &= j \; \text{and} \; i \; \text{is a telomere in} \; G. \end{aligned} $$

### Rank distance

The rank distance *d*(·,·) [33] is defined on $\mathbb {F}^{n \times n}$ via

$$d(A,B) = r(A-B), $$

where *r*(*X*) is the *rank* of the matrix *X*, defined as the dimension of the *image* (or column space) of *X* and denoted im(*X*). The fact that it is a metric follows from the following lemma, which also establishes necessary conditions for equality in the triangle inequality that we are going to use later on.

#### **Lemma 1**

The rank distance *d* is a metric. If *d*(*A,B*)+*d*(*B,C*)=*d*(*A,C*), then there exists a projection matrix *P* such that *B*=*A*+*P*(*C*−*A*).

The proof can be found in the Additional files 1 and 2 of this paper.

We also state a helpful

#### **Corollary 1**

If $x \in \mathbb {R}^{n}$ is a vector such that *Ax*=*Cx* and *d*(*A,B*)+*d*(*B,C*)=*d*(*A,C*), then *Bx*=*Ax* as well. In particular, if *A* and *C* have all row sums equal to 1, so does *B*. In addition, if *A*, *B*, and *C* are genomic and both *A* and *C* have an adjacency (*i,j*), then *B* does, too.

#### *Proof*

The distance condition implies that *B*=*A*+*P*(*C*−*A*) for some *P*, so we can compute *Bx*=*Ax*+*P*(*C*−*A*)*x*=*Ax*+*P*(*Cx*−*Ax*)=*Ax*, so *Bx*=*Ax* as claimed. The second statement follows from the first one by taking *x*=*e*, the vector of *n* 1’s, since *Ae* is the vector containing the row sums of *A*. The third statement comes from the fact that having an adjacency (*i,j*) for genomic *A* is equivalent to *Ae*_*i*_=*e*_*j*_, where *e*_*i*_ is the 0-1 column vector with 1 in position *i* and zeros elsewhere. □

The rank distance between permutation matrices is equivalent to the Cayley distance between the corresponding permutations. We will develop this connection further in the “Permutation matrices” subsection.

## Methods

### Median of 3 matrices

Given three matrices *A,B,C*, the median *M* is defined as a global minimizer of the *score* function

2 $$  d(M;A,B,C) := d(A,M) + d(B,M) + d(C,M).  $$

Since *d* is a metric, we can use symmetry and the triangle inequality to see that the score has a simple lower bound:

$${} \begin{aligned} d(M;A,B,C) &= \frac{d(A,M) + d(M,B)}{2} + \frac{d(B,M) + d(M,C)}{2}\\&\quad + \frac{d(C,M) + d(M,A)}{2} \\ & \geq \frac{d(A,B)}{2} + \frac{d(B,C)}{2} + \frac{d(C,A)}{2}\\& = \frac{1}{2} \left[d(A,B) + d(B,C) + d(C,A)\right], \end{aligned} $$

with equality if and only if

3 $${} \begin{aligned} d(X,M) + d(M,Y) = d(X,Y) \; \text{for any distinct} \; X, Y \in \{A,B,C\}. \end{aligned}  $$

### The first invariant

We now define the first invariant of the median-of-three problem via

$$\beta(A,B,C) := \frac{1}{2} \left[d(A,B) + d(B,C) + d(C,A)\right]. $$

It is easy to see that this is indeed an invariant in the sense that it does not change under permuting of the three matrices, or permuting the rows or the columns of all the matrices in the same way. Namely,

$$\begin{aligned} \beta(A,B,C) &= \beta(A,C,B) = \beta(B,C,A) = \beta(B,A,C)\\ &= \beta(C,B,A) = \beta(C,A,B), \end{aligned} $$

and, for any *n*×*n* permutation matrices *P* and *Q*,

$$\beta(A,B,C) = \beta\left(PAQ^{T},PBQ^{T},PCQ^{T}\right). $$

The fact that *d* is a metric allows us to establish a first (trivial) approximation algorithm with an approximation ratio of $\frac {4}{3}$ [27] - namely, pick the matrix among *A,B,C* with the smallest score. The approximation ratio follows from

$${\kern25pt}\begin{aligned} \min_{X \in \{A,B,C\}} d(X;A,B,C) \leq \frac{1}{3} \sum_{X \in \{A,B,C\}} d(X;A,B,C) \\ = \frac{d(B,A) + d(C,A) + d(A,B) + d(C,B)+ d(A,C) + d(B,C)}{3} = \frac{4}{3} \beta(A,B,C). \end{aligned} $$

We note that any matrix with score *β* is necessarily a median, and that for any matrix *M* that attains this score, we necessarily have

$$\begin{aligned} d(A,M) &= \beta - d(B,C); \; d(B,M)\\ &= \beta - d(A,C); \; d(C,M) = \beta - d(A,B). \end{aligned} $$

However, in the general case, it is not possible to attain the lower bound *β*. For instance, if

$$A = \left(\begin{array}{ll} -1 & 0 \\ 0 & -1 \end{array}\right), B = \left(\begin{array}{ll} 0 & 0 \\ 0 & 0 \end{array}\right), C = \left(\begin{array}{ll} 1 & 0 \\ 0 & 1 \end{array}\right), $$

then *d*(*A,B*)=*d*(*B,C*)=*d*(*C,A*)=2, so *β*=3, but it is easy to check that no matrix is simultaneously at unit rank distance from all three of these matrices, and the minimum score is 4 (attained by, for instance, any diagonal matrix with diagonal entries in {−1,0,1}).

### The second invariant

The second invariant, which was already identified in [23] as playing an important role in the median problem, is the dimension of the “triple agreement” subspace *V*_1_, i.e.:

4 $$  \begin{aligned} \alpha(A,B,C) &:= \text{dim}(V_{1}), \; \text{where} \\ V_{1} &:= \{x \in \mathbb{R}^{n}|Ax = Bx = Cx\}. \end{aligned}  $$

Once again, it is easy to check that it is invariant under permutations of the three matrices, or permutations of the rows or the columns of all the matrices.

### The third invariant

The third invariant, which is a combination of the first two and the dimension of the space, is

5 $$  \delta(A,B,C) := \alpha(A,B,C) + \beta(A,B,C) - n.  $$

We will show in Corollary 3 that it is non-negative for orthogonal arguments. We therefore call it the *deficiency* of *A,B* and *C*, by analogy with the deficiency of a chemical reaction network defined in the work of Horn, Jackson and Feinberg [34]. Our Theorem 1 is thus also a “deficiency zero theorem” for medians of permutations.

### Permutation matrices

Let us now consider the special case of *A,B,C* being permutation matrices. While, as our example showed, the lower bound *β*(*A,B,C*) for the score cannot always be attained, we will show in “Polynomial-time algorithm for a median of three orthogonal matrices” section that the lower bound can always be attained for permutation matrices (and, more generally, orthogonal matrices), meaning that the equality conditions in Eq. (3) can always be satisfied.

#### Integrality of the first invariant

Let us denote by *S*_*n*_ the group of permutations on *n* elements. Pereira Zanetti *et al* [23] have already shown that, for any two permutations *σ* and *τ* in *S*_*n*_, the *transposition distance* *d*_*T*_(*σ*,*τ*), also known as Cayley distance [35] and counting the minimum number of transpositions (switches) needed to transform *σ* into *τ*, equals *d*(*S,T*), where *S* and *T* are the permutation matrices corresponding to *σ* and *τ*, respectively, and *d* is the rank distance.

Let us begin by showing that, if *A,B,C* are permutation matrices, *β*(*A,B,C*) is always an integer; this is also not the case in general, as can be seen in the one-dimensional example of three different scalars, for which *β*(*A,B,C*)=3/2.

To this end, we recall that any permutation *τ*∈*S*_*n*_ can be written as a product of disjoint cycles, uniquely up to order of the cycles and order of the elements within the cycle, provided that fixed points are represented by cycles of length 1. We define the *cycle counter* *c*(*τ*) as the number of disjoint cycles in a disjoint cycle representation of *τ*. For instance, if *τ*=(12)(34)(5), then *c*(*τ*)=3.

##### **Lemma 2**

If *A,B* are permutation matrices corresponding to permutations *ρ*,*σ*, respectively, then $d(A,B) = n - c\left (\rho ^{-1} \sigma \right)$.

##### *Proof*

It was already shown in [23] that *d*(*A,B*)=*d*_*T*_(*ρ*,*σ*), where *d*_*T*_ is the transposition distance. Since *d*_*T*_ is left invariant [35], we have

$$d_{T}(\rho,\sigma) = d_{T}\left(\rho^{-1} \rho, \rho^{-1} \sigma\right) = d_{T}\left(e, \rho^{-1} \sigma\right). $$

It remains to show that the minimum number of transpositions needed to transform a permutation *τ* into the identity is *n*−*c*(*τ*). This follows from the facts that a *k*-cycle needs exactly *k*−1 transpositions to transform into the identity, the total length of all the cycles (including the fixed points) is *n*, and the optimal set of transpositions affects one cycle at a time. □

##### **Corollary 2**

If *A,B* are permutation matrices corresponding to permutations *ρ*,*σ*, respectively, then the kernel of *A*−*B* is spanned by the indicator vectors of the cycles of *ρ*^−1^*σ* (each taking value *1* on the cycle and *0* outside it).

This corollary, which follows from Lemma 2 and the rank-nullity theorem, could also have been deduced directly from the following

##### **Remark 1**

If the permutation matrix *A* corresponds to *ρ*, $Ax = \left [x_{\rho (1)}, \dots, x_{\rho (n)}\right ]^{T}$.

##### **Lemma 3**

If *A,B,C* are permutation matrices, *β*(*A,B,C*) is an integer.

##### *Proof*

Let *ρ*,*σ*,*τ* be the permutations corresponding to *A,B,C*, respectively. From the foregoing discussion, *d*_*T*_(*ρ*,*σ*) is the smallest number of transpositions needed to transform *ρ* into *σ*. Note that transforming *ρ* into *σ* and then *σ* into *τ* also transforms *ρ* into *τ*. In addition, it is known that the number of transpositions needed to transform one permutation into another has a fixed parity that only depends on the *signs* of the permutations [35]. Therefore

$$\begin{aligned} d(A,B) + d(B,C) + d(C,A) = d_{T}(\rho,\sigma) + d_{T}(\sigma,\tau) + d_{T}(\rho,\tau) \equiv\\ \equiv d_{T}(\rho,\sigma) + d_{T}(\sigma,\tau) + \left[d_{T}(\rho,\sigma) + d_{T}(\sigma,\tau)\right] \equiv 0 \mod 2, \end{aligned} $$

so that *β*(*A,B,C*) is indeed an integer.

An alternative proof can be obtained by noting that $(-1)^{d_{T}(\rho,\sigma)} = \det (A^{-1}B)$, where *A,B* are the permutation matrices for *ρ*,*σ* respectively; therefore

$$\begin{aligned} &(-1)^{d_{T}(\rho,\sigma) + d_{T}(\sigma, \tau) + d_{T}(\tau, \rho)} \,=\, \det\left(A^{-1}B\right) \det\left(B^{-1}C\right) \!\det\left(C^{-1}A\right) =\\ &= \det\left(\left(A^{-1}B\right)\left(B^{-1}C\right)\left(C^{-1}A\right)\right) = \det\left(A^{-1}\left(BB^{-1}\right)\left(CC^{-1}\right)A\right) =\\ &= \det(I) = 1 \implies d_{T}(\rho,\sigma) + d_{T}(\sigma, \tau) + d_{T}(\tau, \rho) \equiv 0 \mod 2. \end{aligned} $$

□

In Additional file 1 there is a proof that this result extends to orthogonal matrices *A,B,C* as well.

#### Fast computation of the invariants

We now show how to compute the invariants *α*, *β* and *δ* in *O*(*n*) time given the three permutations *ρ*,*σ*,*τ* represented by *A,B,C*, respectively. For *β*, it suffices to use the identity

$${} \beta(A,B,C) = \frac{1}{2} \bigl(3n - \left[c\left(\rho^{-1} \sigma\right) + c\left(\sigma^{-1} \tau\right) + c\left(\tau^{-1} \rho\right)\right] \bigr), $$

obtained using Lemma 2 and the definition of *β*. Permutations can be multiplied and inverted in *O*(*n*) time using standard algorithms, and their cycles can be counted in *O*(*n*) time by using their graph representation (with a directed edge from *i* to *π*(*i*) for every 1≤*i*≤*n*) and identifying the weakly connected components. Therefore, the computation of *β*(*A,B,C*) takes *O*(*n*) time overall.

For *α*, we note that, by Corollary 2,

$$\begin{aligned} x \in V_{1} \iff Ax = Bx = Cx \iff Ax = Bx \; \text{and} \; Bx = Cx \iff\\ \iff x \; \text{is constant on the cycles of} \; \rho^{-1} \sigma \; \text{and on the cycles of} \; \sigma^{-1} \tau. \end{aligned} $$

The computation of *ρ*^−1^*σ* and *σ*^−1^*τ* is the same as the one performed for computing *β*, and the dimension of *V*_1_ is then equal to the number of weakly connected components of the union of their graph representations described above.

Indeed, if *C*_1_,*C*_2_ are 2 disjoint weakly connected components of the graph representation of *ρ*^−1^*σ* and there is an edge between *C*_1_ and *C*_2_ in the graph representation of *σ*^−1^*τ*, then any vector *x*∈*V*_1_ must be constant on $C_{1} \cup C_{2}$. By iterating this reasoning, we conclude that *α* is precisely the number of weakly connected components of the union of the graph representations of *ρ*^−1^*σ* and *σ*^−1^*τ*. Each graph can be computed in *O*(*n*) time, and so can their union and its connected components, so computing *α* also requires *O*(*n*) time overall.

Finally, *δ* can be computed from *α* and *β* in constant time using its definition.

#### Subspace dimensions in terms of invariants

Pereira Zanetti *et al* [23] showed how to decompose the space $\mathbb {R}^{n}$ into a direct sum of five subspaces, and expressed their median candidates using the projection operators of these subspaces. We now show how to express the dimensions of these subspaces using the invariants *α*, *β* and *δ*, and deduce a sufficient condition for their median candidate to be a true median. Readers familiar with this paper will recognize our use of the dot notation for partitions introduced there (e.g.,.*AB*.*C*). However, all the subspaces needed here are defined here as well, so the exact meaning of this notation is not relevant in our context.

The “triple agreement” subspace *V*_1_=*V*(.*A*.*B*.*C*.) is defined in Eq. (4), and is the subspace of all vectors on which all three matrices agree. Its dimension is *α*, by definition.

The subspace $V_{2} := V(.AB.C.) \cap V_{1}^{\perp }$ is defined via *V*_1_ and the subspace

$$V(.AB.C) := \{x \in \mathbb{R}^{n}|Ax = Bx\}. $$

The dimension of *V*(.*AB*.*C*) is precisely *c*(*ρ*^−1^*σ*), where *ρ* and *σ* are the permutations corresponding to *A* and *B*, respectively. This follows from Corollary 2 which tells us that

$$\begin{aligned} Ax = Bx \iff A^{-1}Bx = x \iff x \; \text{is constant on every cycle of} \; \rho^{-1} \sigma. \end{aligned} $$

Since $V_{1} \subseteq V(.AB.C)$, it follows that a basis of *V*_1_ can be extended to a basis of *V*(.*AB*.*C*) with vectors orthogonal to those spanning *V*_1_, so that

$$\begin{aligned} \text{dim}(V_{2}) &= \text{dim}(V(.AB.C.) \cap V_{1}^{\perp})\\ &= \text{dim}(V(.AB.C.) - \text{dim}(V_{1}) = c(\rho^{-1} \sigma) - \alpha. \end{aligned} $$

We can apply a similar reasoning to the subspaces $V_{3} := V(.A.BC.) \cap V_{1}^{\perp }$ and $V_{4} := V(.AC.B) \cap V_{1}^{\perp }$, where $V(.A.BC.) := \{x \in \mathbb {R}^{n}|Bx = Cx\}$ and $V(.AC.B) := \{x \in \mathbb {R}^{n}|Cx = Ax\}$, to get

$$\begin{aligned} \text{dim}(V_{2}) &= c\left(\rho^{-1} \sigma\right) - \alpha; \; \text{dim}(V_{3})\\ &= c\left(\sigma^{-1} \tau \right) - \alpha; \; \text{dim}(V_{4}) = c\left(\tau^{-1} \rho\right) - \alpha. \end{aligned} $$

It was shown by Pereira Zanetti et al. [23] that

6 $$  \mathbb{R}^{n} = V_{1} \oplus V_{2} \oplus V_{3} \oplus V_{4} \oplus V_{5},  $$

where *V*_5_ is the subspace orthogonal to the sum of the other four subspaces, and the ⊕ notation represents a direct sum, i.e. $V_{i} \cap V_{j} = \{0\}$ whenever 1≤*i*<*j*≤5.

Since *V*_5_:=*V*(*ABC*) is the last term in the direct sum decomposition of $\mathbb {R}^{n}$, we get that

$${} \begin{aligned} \text{dim}(V_{5}) &= n - \sum_{i=1}^{4} \text{dim}(V_{i}) = n + 2 \alpha - \left(c\left(\rho^{-1} \sigma\right)\right. \\&\qquad \qquad +\left. c\left(\sigma^{-1} \tau\right) + c\left(\tau^{-1} \rho\right)\right) =\\ &= n + 2 \alpha(A,B,C) - (3n - 2\beta(A,B,C))\\& = 2 (\alpha + \beta\ - n) = 2 \delta(A,B,C). \end{aligned} $$

From this, we immediately deduce the following

##### **Corollary 3**

*δ*(*A,B,C*)≥0 for permutation matrices *A,B,C*, with equality if and only if *V*_5_={0}.

In addition, we can now combine this with the expression in [23] for the score of their median candidates *M*_*A*_,*M*_*B*_,*M*_*C*_ (obtained by taking the common value on each vector in an “agreement subspace”, *V*_1_ through *V*_4_, and the value corresponding to multiplication by *A*, *B* or *C*, respectively, on the remaining subspace *V*_5_) to get

$$\begin{aligned} d(M_{A};A,B,C) &= 2 \text{dim}(V_{5}) + \text{dim}(V_{2}) + \text{dim}(V_{3}) + \text{dim}(V_{4})\\ &= n - \text{dim}(V_{1}) + \text{dim}(V_{5})\\ &= n - \alpha + 2 (\alpha + \beta - n)\\ &= \beta + (\alpha + \beta - n)\\ &= \beta + \frac{1}{2} \text{dim}(V_{5})\\ &= \beta + \delta. \end{aligned} $$

As expected, the median *M*_*A*_ achieves the lower bound if and only if *V*_5_={0}.

#### A new algorithm

Next we introduce a new algorithm that can be used to identify another candidate median, that in general differs from the candidates *M*_*A*_,*M*_*B*_,*M*_*C*_ identified in previous work [23]. Although we are not able to prove any approximation ratio on its performance, it does allow us to establish the uniqueness of the median in the special case of equality in Corollary 3, and gain insight into why this case is special, in addition to obtaining a faster *O*(*n*^2^) running time for it.

To this end, let us consider the necessary conditions from Lemma 1 that must be satisfied in order for the matrix *M* to attain the lower bound *β*:

7 $$ M = A + S(B-A) = B + T(C-B) = C + U(A-C),  $$

where *S,T,U* are some projection matrices. This triple equality follows from the facts that Eq. (3) must be satisfied for each pair among *A,B* and *C*.

Let us count the independent equations and non-redundant unknowns in this system. We note that it suffices to consider the equivalent system

8 $$  \begin{aligned} A + S(B-A) &= B + T(C-B) \iff \\ A - B &= T(C-B) - S(B-A); \\ B + T(C-B) &= C + U(A-C) \iff \\ B - C &= U(A-C) - T(C-B), \end{aligned}  $$

since the third equality automatically follows from the first two. Furthermore, we will not enforce the condition of *S,T,U* being projection matrices, since a projection matrix is defined by *P*^2^=*P* and this results in a set of conditions quadratic in the entries of *P*.

Consider the effect of multiplying a matrix *S* by a permutation matrix *A* corresponding to the permutation *ρ*. It is easy to see that this results in permuting the columns of *S* according to *ρ*, so that, denoting by *s*_*i*_ the *i*-th column of *S*, we get $SA = \left [s_{\rho (1)} s_{\rho (2)} \dots s_{\rho (n)}\right ]$. Therefore, the *i*-th column of *S*(*B*−*A*) will simply be the difference between *s*_*ρ*(*i*)_ and *s*_*σ*(*i*)_. For each cycle *C* of *ρ*^−1^*σ*, the corresponding columns of *S*(*B*−*A*) will add up to 0.

Thus, changing variables to the “difference variables” of the form

9 $$  s'_{i} := s_{\rho(C[i])} - s_{\sigma(C[i])},  $$

where $C\left [i\right ]$ denotes the *i*-th element in the cycle *C*, we can see that *S*(*B*−*A*) will have precisely *n*−*c*(*ρ*^−1^*σ*) linearly independent columns, and one column per cycle will be linearly dependent on the others in the same cycle. By applying the same argument to *T*(*C*−*B*) and *U*(*A*−*C*), we get a grand total of

$$n - c\left(\rho^{-1} \sigma\right) + n - c\left(\sigma^{-1} \tau\right) + n - c\left(\tau^{-1} \rho\right) = 2\beta(A,B,C) $$

linearly independent (non-redundant) columns, each containing *n* free variables.

We note that the system of Eq. (8), rewritten with respect to the non-redundant difference variables, splits into *n* independent linear systems, one per row, with identical left-hand sides and only differing by their right-hand sides:

10 $$  \begin{aligned} \sum\limits_{i = 1}^{d(A,B)} p_{i} s_{i} + \sum\limits_{i=1}^{d(B,C)} q_{i} t_{i} = a_{ji} - b_{ji}\; ; \; \sum\limits_{i=1}^{d(C,A)} r_{i} u_{i} - \sum\limits_{i=1}^{d(B,C)} q_{i} t_{i} = b_{ji} - c_{ji}, \end{aligned}  $$

where the *p*_*i*_,*q*_*i*_,*r*_*i*_ are coefficients that only depend on the column (variable) index *i*, but not on the row *j*.

Next we count the linearly independent equations within each system. In the first half of the system (with right-hand sides coming from the *j*-th row of *A*−*B*), linear dependence between the left-hand sides of the equations can only arise from vectors *y* such that

$$T(C-B)y = 0 = S(B-A)y \; \forall \; S,T, $$

since *S* and *T* represent the variables in the system and those variables are distinct. Similarly, in the second half of the system, they can only arise from vectors *y* such that

$$U(A-C)y = 0 = T(C-B)y \; \forall \; T,U, $$

since *T* and *U* represent the variables in the system and those variables are distinct. But then, for any such vector *y* it must be the case that

$$Ay = By = Cy \iff y \in V_{1}, $$

meaning that there are exactly *α*(*A,B,C*) dependence relationships between the left-hand sides in each of the half-systems since that is the dimension of the subspace *V*_1_.

Furthermore, all such dependence relationships lead to the tautology 0=0 rather than the contradiction 0=1, because every row of *A*−*B* and *B*−*C* is orthogonal to any *y*∈*V*_1_, by definition of *V*_1_. Lastly, the two half-systems are linearly independent from one another since the condition on their linear dependence is subsumed by the condition of the linear dependence within the half-systems; more precisely, since the *t*-variables in the first half-system appear with coefficients that are exactly the negative of the coefficients in the second half-system, a linear dependence relationship between the half-systems would have to arise from a vector *y* such that

$$\begin{aligned} &U(A-C)y = 0 = S(B-A)y \; \forall \; S,U \iff\\ &Ay = By = Cy \iff y \in V_{1}. \end{aligned} $$

It follows that after eliminating the redundancies in each half-system, exactly *β* variables and *n*−*α* equations remain. Since *α*+*β*≥*n* the system is always under-constrained except in the special case of *δ*=*α*+*β*−*n*=0, in which it has a unique solution (since the remaining equations are linearly independent).

In the special case when *δ*=0, we can furthermore choose to eliminate precisely those redundant equations that contain the “composite” difference variables of the form $- s_{1} - s_{2} - \dots - s_{k}$, corresponding to a cycle of length *k*+1 in the appropriate permutation. In this way, the remaining equations will only have two active variables (with non-zero coefficients) each, so the algorithm by Aspvall and Shiloach [36] can be applied to solve the resulting system in *O*(*n*) time. It follows that the algorithm will only require *O*(*n*^2^) time to find the median in the special case.

Although the system is under-constrained outside of the special case, we can use ideas from the field of *compressed sensing* [37] to find a solution that is likely to be sparse, and hence hopefully low rank. Namely, we seek the solution containing as many zeros as possible. While this is a hard problem in general, many instances are solvable by using the *L*_1_ norm minimization, which can be achieved by using linear programming. The appropriate linear program becomes

$$\min \sum_{i} y_{i} \; \text{subject to} \; -y_{i} \leq x_{i} \leq y_{i} \; \forall \; i \; \text{and} \; Ux = b, $$

where *Ux*=*b* is the original system of equations, and the *y*_*i*_ serve as the absolute values of the *x*_*i*_ whose sum is to be minimized. Such linear programs can be readily solved using existing off-the-shelf solvers such as lpsolve [38] or CPLEX [39].

#### An example of the algorithm

To clarify the procedure, we now illustrate the running of our algorithm on *ρ*=(12)(34),*σ*=(13)(24),*τ*=(14)(23), with *n*=4. First, we note that the product of any two of these equals the third, and they are each their own inverse. Hence we can compute

$${} \begin{aligned} \beta(A,B,C) &= \frac{1}{2} \bigl(3n - \left[c\left(\rho^{-1} \sigma\right) + c\left(\sigma^{-1} \tau\right) + c\left(\tau^{-1} \rho\right)\right] \bigr)\\ &= \frac{1}{2} \left[12 - 3 \cdot 2\right] = 3 \end{aligned} $$

and

$$\alpha(A,B,C) = 1 $$

since the union of the graphs of *ρ*^−1^*σ*=*τ* and *σ*^−1^*τ*=*ρ* has a unique weakly connected component. Therefore, *α*(*A,B,C*)+*β*(*A,B,C*)=*n* and, as we will prove below, the algorithm will in fact produce the unique median of *A,B,C*.

We start by forming the equations in system (8):

$$\begin{array}{*{20}l} A - B &= \left[t_{4} - t_{3}, t_{3} - t_{4}, t_{2} - t_{1}, t_{1} - t_{2}\right]\\&\quad - \left[s_{3} - s_{2}, s_{4} - s_{1}, s_{1} - s_{4}, s_{2} - s_{3}\right]\\ B - C &= \left[u_{2} - u_{4}, u_{1} - u_{3}, u_{4} - u_{2}, u_{3} - u_{1}\right]\\&\quad - \left[t_{4} - t_{1}, t_{3} - t_{4}, t_{2} - t_{1}, t_{1} - t_{2}\right]. \end{array} $$

We now define the “difference variables”

$$\begin{aligned} &s'_{1} := s_{3} - s_{2}; s'_{2} := s_{4} - s_{1}; t'_{1} := t_{4} - t_{3}; t'_{2} := t_{2} - t_{1};\\ &u'_{1} := u_{2} - u_{4}; u'_{2} := u_{1} - u_{3}, \end{aligned} $$

and express our system in terms of those:

$$\begin{array}{*{20}l} A - B &= \left[t'_{1}, -t'_{1}, t'_{2}, -t'_{2}\right] - \left[s'_{1}, s'_{2}, -s'_{2}, -s'_{1}\right]\\ B - C &= \left[u'_{1}, u'_{2}, -u'_{1}, -u'_{2}\right] - \left[t'_{1}, -t'_{1}, t'_{2}, -t'_{2}\right]. \end{array} $$

For convenience we will use the same name (without row superscripts) for the variables in each row, to emphasize that the system of equations for each row has the same left-hand side. For row *j* it has 2*n*=8 equations that read:

11.1 $$\begin{array}{*{20}l} t'_{1} - s'_{1} = A_{j1} - B_{j1} \end{array} $$

11.2 $$\begin{array}{*{20}l} -t'_{1} - s'_{2} = A_{j2} - B_{j2} \end{array} $$

11.3 $$\begin{array}{*{20}l} t'_{2} + s'_{2} = A_{j3} - B_{j3} \end{array} $$

11.4 $$\begin{array}{*{20}l} -t'_{2} + s'_{1} = A_{j4} - B_{j4}  \end{array} $$

11.5 $$\begin{array}{*{20}l} u'_{1} - t'_{1} = B_{j1} - C_{j1} \end{array} $$

11.6 $$\begin{array}{*{20}l} u'_{2} + t'_{1} = B_{j2} - C_{j2} \end{array} $$

11.7 $$\begin{array}{*{20}l} -u'_{1} - t'_{2} = B_{j3} - C_{j3} \end{array} $$

11.8 $$\begin{array}{*{20}l} -u'_{2} + t'_{2} = B_{j4} - C_{j4}  \end{array} $$

As expected from our counting argument above, the only linear dependencies here are that the first 4 equations add up to 0 and the second 4 equations add up to 0 (both their left-hand sides and their right-hand sides), so after eliminating, say, Eqs. (11.4) and (11.8), we end up with a consistent and non-redundant system of 2(*n*−*α*)=6 equations in 2*β*=6 unknowns, which therefore has a unique solution. We illustrate this system for the first row, *j*=1, since it looks identical for all the other rows except for changes in its right-hand side.

12.1 $$\begin{array}{*{20}l} t'_{1} - s'_{1} = 0 \end{array} $$

12.2 $$\begin{array}{*{20}l} -t'_{1} - s'_{2} = 1 \end{array} $$

12.3 $$\begin{array}{*{20}l} t'_{2} + s'_{2} = -1 \end{array} $$

12.4 $$\begin{array}{*{20}l} u'_{1} - t'_{1} = 0 \end{array} $$

12.5 $$\begin{array}{*{20}l} u'_{2} + t'_{1} = 0 \end{array} $$

12.6 $$\begin{array}{*{20}l} -u'_{1} - t'_{2} = 1 \end{array} $$

Since each equation has exactly two variables, we can use the method of [36] to solve them in *O*(*n*) time, which means that the total time to solve the system for all the *n* rows is *O*(*n*^2^). In the case of this system, we see that the solution for the first row is

$${} s'_{1} = -\frac{1}{2}, s'_{2} = -\frac{1}{2}, t'_{1} = -\frac{1}{2}, t'_{2} = -\frac{1}{2}, u'_{1} = -\frac{1}{2}, u'_{2} = \frac{1}{2}. $$

After solving the system for all the other rows in the same way, we conclude that the unique median of *A,B,C* in this case is $\frac {1}{2} J - I$, where *J*=*ee*^*T*^ is the matrix of all 1’s and *I* is the identity matrix. In particular, this example shows that the unique median of three genomic matrices may not itself be genomic (or even binary).

#### Example of the compressed sensing approach

We now consider a different example, one which does not fall into the special case *α*+*β*=*n*. We take *n*=3 and *ρ*=(12),*σ*=(13),*τ*=(23). In this case, we have *d*(*A,B*)=*d*(*B,C*)=*d*(*C,A*)=2 so *β*(*A,B,C*)=3 and *α*(*A,B,C*)=1. We know that the system of Eq. (8) will be under-constrained in this case. We start by forming this system of equations:

$$\begin{aligned} &A - B = \left[t_{1} - t_{3}, t_{3} - t_{2}, t_{2} - t_{1}\right] - \left[s_{3} - s_{2}, s_{2} - s_{1}, s_{1} - s_{3}\right]\\ &B - C = \left[u_{2} - u_{1} u_{1} - u_{3} u_{3} - u_{2}\right] - \left[t_{1} - t_{3}, t_{3} - t_{2}, t_{2} - t_{1}\right] \end{aligned} $$

and introduce the difference variables

$$\begin{aligned} &s'_{1} := s_{3} - s_{2}, s'_{2} := s_{2} - s_{1}, t'_{1} := t_{1} - t_{3}, t'_{2} := t_{3} - t_{2},\\ &u'_{1} := u_{2} - u_{1}, u'_{2} := u_{1} - u_{3} \end{aligned} $$

to rewrite it as 3 systems (one for each row) of the form

13.1 $$\begin{array}{*{20}l} t'_{1} - s'_{1} = A_{1j} - B_{1j} \end{array} $$

13.2 $$\begin{array}{*{20}l} t'_{2} - s'_{2} = A_{2j} - B_{2j} \end{array} $$

13.3 $$\begin{array}{*{20}l} -\left(t'_{1} + t'_{2}\right) + \left(s'_{1} + s'_{2}\right) = A_{3j} - B_{3j}  \end{array} $$

13.4 $$\begin{array}{*{20}l} u'_{1} - t'_{1} = B_{1j} - C_{1j} \end{array} $$

13.5 $$\begin{array}{*{20}l} u'_{2} - t'_{2} = B_{2j} - C_{2j} \end{array} $$

13.6 $$\begin{array}{*{20}l} -\left(u'_{1} + u'_{2}\right) + \left(t'_{1} + t'_{2}\right) = B_{3j} - C_{3j}  \end{array} $$

As in the previous example, we can eliminate the redundant Eqs. (13.3) and (13.6) from each system, leaving us with a total of 4 equations in 6 variables.

Let us now show the compressed sensing formulation for this system for row *j*=1. We get the linear program

$$\begin{array}{*{20}l} &{} \text{minimize} \; y_{1} + y_{2} + y_{3} + y_{4} + y_{5} + y_{6}\\ &{} \text{subject to} \\ &{} - \left[y_{1}, y_{2}, y_{3}, y_{4}, y_{5}, y_{6}\right] \leq \left[s'_{1}, s'_{2}, t'_{1}, t'_{2}, u'_{1}, u'_{2}\right]\\ &{} \leq \left[y_{1}, y_{2}, y_{3}, y_{4}, y_{5}, y_{6}\right]\\ &{} t'_{1} - s'_{1} = 0; \quad t'_{2} - s'_{2} = 1; \quad u'_{1} - t'_{1} = -1; \quad u'_{2} - t'_{2} = 0. \end{array} $$

The optimal solution to this linear program is

$$s'_{1} = 0, s'_{2} = -1, t'_{1} = 0, t'_{2} = 0, u'_{1} = -1, u'_{2} = 0. $$

By repeating this for the other two rows we obtain the solution $M = \left [0 \; 0 \; e\right ]$, which unfortunately yields a suboptimal score of 6, whereas the optimal solutions, given by the identity matrix, either of the 3-cycles (123) or (132), or a subset of the affine combinations of those matrices, yield a score of *β*=3. This shows that the compressed sensing approach is not guaranteed to be optimal, or even better than the algorithm that picks the best “corner" option among *A,B,C*.

#### Proof of uniqueness for the special case

We now prove that if *δ*(*A,B,C*)=0, then there is a unique median, and both the *O*(*n*^3^) algorithm by Pereira Zanetti *et al* [23] as well as our *O*(*n*^2^) algorithm proposed here correctly identify it.

##### **Theorem 1**

Suppose that *A,B,C* are permutations with *δ*(*A,B,C*)=0. Then the median is unique, and it is found by our algorithm.

##### *Proof*

By the calculations in [23] recapitulated in Corollary 3, the median *M*_*A*_ achieves the lower bound *β*. Furthermore, by the calculations in the analysis of the system (8), we see that there exists a unique matrix *M* that simultaneously satisfies the necessary conditions for attaining the lower bound *β*. Since *M*_*A*_ attains this lower bound, *M*_*A*_ also satisfies these necessary conditions; by uniqueness, *M*_*A*_=*M*, so our algorithm also finds a median, and it is unique. □

#### Rarity of the special case

We now use some asymptotic results from analytic combinatorics to show that the probability of three random genomic matrices satisfying the optimality conditions in Corollary 3 tends to 0 as *n* increases. Recall that an *involution* is a permutation that is its own inverse; this is precisely the class of permutations defined by genomic matrices. We begin by restating, without proof, the following result from [40]:

##### **Theorem 2**

If *σ* and *τ* are random involutions, then the mean number of cycles of *στ* is $\sqrt {n} + \frac {1}{2} \log n + O(1)$. If *σ* and *τ* are constrained to be fixed-point free, then the distribution of the number of cycles of *στ* is asymptotically normal with mean $\log n$ and variance $2 \log n$.

Now we can immediately conclude the following

##### **Corollary 4**

If *A,B,C* are the genomic matrices corresponding to random involutions (respectively random involutions with no fixed points, i.e. telomeres), then $\beta \sim \frac {3}{2} (n - \sqrt {n})$ or $\beta \sim \frac {3}{2} (n - \log n)$, respectively. In particular, the probability of these matrices satisfying the optimality conditions in Corollary 3 tends to *0* as *n* increases.

**Fig. 6:**
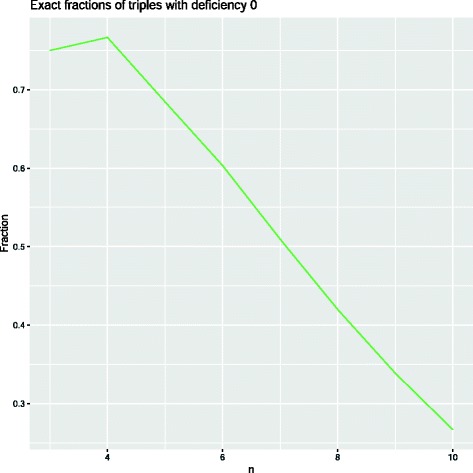
Exact fractions of distinct triples of involutions *A,B,C* of size *n* satisfying *δ*(*A,B,C*)=0, for small values of *n*

However, the result from analytic combinatorics does not tell us anything about the rate of convergence of this probability to 0. For this reason, we decided to investigate the fraction of all distinct triples of genomic matrices of dimension *n* for which *δ*=0. Due to the combinatorial growth of the number of involutions, we were only able to compute this exactly for *n*≤10 These results are shown in Fig. 6 below. It is remarkable that the fraction is already just above $\frac {1}{4}$ for *n*=10.

### Polynomial-time algorithm for a median of three orthogonal matrices

Permutation matrices are orthogonal matrices. While a median of permutation matrices is not always a permutation, there is at least one median for each triple of orthogonal matrices that is orthogonal and satisfies the lower bound *β*. We present here a proof of this fact and a polynomial-time algorithm to find such a median.

The idea is to “walk towards the median”, as follows. Start with orthogonal matrices *A*, *B*, and *C*. If *B* is on a shortest path between *A* and *C*, then *B* itself is the orthogonal median that satisfies the lower bound. Otherwise, we have *d*(*A,B*)+*d*(*B,C*)>*d*(*A,C*). From this inequality we are able to derive that $\text {dim}(\text {im}(A-B) \cap \text {im}(C-B)) > 0$, and therefore there is a non-zero vector $u \in \text {im}(A-B) \cap \text {im}(C-B)$. With this vector we construct a rank 1 matrix *H*=−2*uu*^*T*^*B*/*u*^*T*^*u* which, added to *B*, produces an orthogonal matrix *B*+*H* that is on a shortest path between *B* and *A*, and also on a shortest path between *B* and *C*. The matrix *B*+*H* is then one step closer to the median than *B* is. Proceeding in this way, with *B*+*H* now replacing *B*, we repeat the procedure until we hit a shortest path between *A* and *C*, reaching an orthogonal median that satisfies the lower bound for the three input matrices.

The corresponding pseudo-code appears in Algorithm 1.



This algorithm clearly runs in polynomial time, since the recursion is no more than *n* levels deep, and each recursive call adds no more than a cubic number of steps to the total running time, which is then *O*(*n*^4^). To prove that this algorithm is correct, we need a series of results, as follows:

1. We need to prove that if *d*(*A,B*)+*d*(*B,C*)>*d*(*A,C*), then $\text {dim}(\text {im}(A-B) \cap \text {im}(C-B)) > 0$;
2. We need to prove that if *u* is a non-zero vector in $\text {im}(A-B) \cap \text {im}(C-B)$, then the matrix *H*=−2*uu*^*T*^*B*/*u*^*T*^*u* represents a step from *B* towards both *A* and *C* simultaneously;
3. We need to prove that any median of *A*, *B*+*H*, and *C* that satisfies the lower bound *β*(*A,B*+*H,C*) is also a median of *A*, *B*, and *C* that satisfies the lower bound *β*(*A,B,C*) as well;
4. We need to prove that *B*+*H* is orthogonal.

We prove each one of these partial results in Additional file 1.

## Results

We tested our algorithms on two datasets - first, a simulated one obtained by applying rearrangement operations to a starting genome, and second, a real one obtained by taking three genomes at a time from a family of plants. In this section, we describe the performance of our algorithms as well as our observations. All the data and results can be accessed at *10.5281/zenodo.1202505*.

### Implementation

For the implementation of the exact *O*(*n*^2^) and the heuristic compressed sensing algorithms we use the R statistical computing language [41] as well as the CPLEX linear programming solver [39], with which we interface via the command line. Specifically, our program first computes the invariants *α*, *β* and *δ*, and then branches into either an exact solution if *δ*=0, or the compressed sensing heuristic if not. In the latter case, R writes the linear program for finding the solution of system (8) of minimum *L*_1_ norm into a file, then CPLEX solver processes this file, and R parses the solution to obtain the median candidate.

We use the *igraph* package [42] to quickly compute the invariants as well as the cycle decompositions of the permutations involved in the system (8). The cycle decompositions allow us to decide which variables to include in the system, and which equations to exclude to make it non-redundant. The resulting system always has 2(*n*−*α*) equations in 2*β* variables. In order to try to make the system as sparse as possible even when *δ*>0, we make the variable corresponding to the last (highest) element of each cycle non-basic by expressing it in terms of the others, as per Eq. (9). Furthermore, we eliminate the equation corresponding to the last (highest) element of each connected component in the union graph defining *α*; since each such connected component is a disjoint union of cycles (of each of the three permutations *ρ*^−1^*σ*,*σ*^−1^*τ*,*τ*^−1^*ρ*), this guarantees that fewer composite variables remain present, leading to a sparser system (8).

In the special case *δ*=0, we use the *solve* function from the *Matrix* package [43], and do not implement the linear-time algorithm by Aspvall and Shiloach [36]. Therefore, strictly speaking our implementation is currently not guaranteed to run in *O*(*n*^2^) time despite having a sparse coefficient matrix with all non-zero coefficients being ±1. However, since *solve* is able to take advantage of the sparsity of the system, the special case runs extremely efficiently even for the largest input size, *n*=500.

For large problem sizes, especially those with *n*=500, the linear program solved by the compressed sensing heuristic is highly degenerate. This causes the solver to be relatively slow, occasionally requiring nearly 200,000 iterations (for *n*=500 and high values of *r*), and compels it to introduce a perturbation of the problem in order to make it less degenerate. Nevertheless, even in the presence of this degeneracy the solution ends up being reasonably efficient, as discussed below.

We implemented the second algorithm, which finds a median exactly for any orthogonal input matrices, in GNU Octave version 3.8.1 [44], to take advantage of its ability to quickly compute the nullspace of a matrix. The formula

$$\text{Null}\left(\left[\left(\text{Null}\left(A^{T} - B^{T}\right)\right)^{T}; \left(\text{Null}\left(C^{T} - B^{T}\right)\right)^{T}\right]\right), $$

where Null(·) denotes a basis of the nullspace of its argument, and “ ;” denotes the “stacking up” of two matrices, provides a fast way of computing the intersection $\text {im}(A-B) \cap \text {im}(C-B)$. Their equivalence follows from the fact that

$$\begin{aligned} (\text{im}(A-B) \cap \text{im}(C-B))^{\perp} &= \text{im}(A-B)^{\perp} + \text{im}(C-B)^{\perp}\\ &= \ker\left(A^{T} - B^{T}\right) + \ker\left(C^{T} - B^{T}\right), \end{aligned} $$

where we used $(\text {im} X)^{\perp } = \ker \left (X^{T}\right)$ in the last step. However, this formula may be numerically unstable at times (see Additional file 2), incorrectly giving a subspace equal to {0} when *B* is not between *A* and *C*. In these cases, we resort to an alternative computation for the intersection subspace:

$$\text{Orth}((A-B) * \text{Null}\left(\left[A-B \;\; C-B\right]\right)(1:n,:)), $$

which is slightly slower, but has less Null computations, and is able to return the correct subspace when the primary formula does not work. In our 540 simulated examples, the algorithm resorted to the alternative formula in just 6 cases, one of which is reported in Additional file 2. Once again, we only use this algorithm when *δ*>0, preferring the optimal *O*(*n*^2^) algorithm when *δ*=0. For the orthogonal algorithm experiment, we used a tolerance of *ε*=10^−6^ in all rank computations.

In order to speed up the algorithm we replaced this nullspace computation by the simpler one involving the identification of any pair *i,j* that is in the same cycle of *AB*^−1^ and also in the same cycle of *CB*^−1^ whenever *A,B,C* are permutation matrices, when such a pair exists. Such a pair can be computed in *O*(*n*) time using the graph representations of *AB*^−1^ and *CB*^−1^. It can furthermore be shown that in this case, the resulting rotated version of *B* will remain a permutation matrix (it is in fact simply *B* composed with the transposition (*ij*)). This simple optimization provides important speed-ups and also improves the numerical stability.

### Numerical stability

Computing the score of the median candidates, as defined in Eq. (2), requires a rank computation, which is known to be numerically challenging [45]. In fact, Pereira Zanetti *et al* report that despite all their median candidates being expected to have the same score, this is not true in practice for random permutation inputs in about 10% of the cases when using GNU Octave or MATLAB [23].

In order to circumvent this challenge we adopt several measures. First, we use the combinatorial expression from Corollary 3 for the score of the median candidates proposed in [23] in all our comparisons, which does not require any rank computation, only a graph-based analysis of the underlying permutations. Second, to score the candidate median produced by the compressed sensing algorithm presented here, we use the *rankMatrix* function with the *QR* decomposition method from the *Matrix* package [43], with a tolerance of *ε*=10^−12^. Lastly, we round any entry of the median that happens to be within *ε* of an integer (0 or ±1) to this integer. While we cannot be completely sure that this bypasses all numerical stability issues, we observe that in all instances with *δ*=0, on which our algorithm should produce a median achieving the lower bound *β*, this is indeed the case.

The exact *O*(*n*^4^) algorithm also converges to a correct answer on all 540 inputs.

### Simulated dataset

The simulated dataset consists of a collection of 540 genomic inputs, ranging in size from 6 to 250 genes (i.e. *n*=12 to *n*=500), exactly as in the simulated dataset used by Pereira Zanetti *et al* [23]. We generate the simulated instances as follows. We start with a unichromosomal linear genome with *n*/2 genes and apply a random number between $\frac {rn}{2}$ and $\frac {3rn}{2}$ DCJ operations [9] to obtain each of the three input genomes, where *r* is a fraction between 0 and 1 (we refer to *r* as the *rearrangement rate*). After that we cut any circular chromosomes so that the resulting instance has three multi-chromosomal linear genomes. We use values of *r* ranging between 0.05 and 0.3, as higher values of *r* may require a distance correction and lead parsimony-based methods to produce incorrect results [46]. For each setting of *n* and *r* we generate 10 instances, and report the averages.

First, we observe that the exact *O*(*n*^2^) algorithm for the case *δ*=0 is extremely fast, requiring less than 45 s in total for all the 473 inputs (or 87.5%) that belong to it, i.e. less than 0.1 s per instance on average. The compressed sensing algorithm is somewhat slower, requiring a total of 105 s for the 67 inputs that it ran on, for an average of just over 1.5 s per instance. However, the time for all but the largest instances is in fact dominated by writing and reading the linear program file, not the actual solution. For instance, reading the file and solving the linear program each take CPLEX around 1.5 s when *n*=500. In short, producing the median candidate using our method is extremely efficient relative to both the *O*(*n*^3^) computation proposed by Pereira Zanetti et al. [23] as well as the exact and heuristic methods they compared it to.

Second, we observe that the vast majority of the inputs produce median candidates that are genomic matrices. More specifically, only 12 out of 540 outputs contain fractional values (and all of these are actually optimal as they fall into the case *δ*=0); these fractional values are $\pm \frac {1}{2}, \pm \frac {1}{3}, \frac {2}{3}, \pm \frac {1}{4}$ and $\frac {3}{4}$. The remaining 528 out of 540 outputs contain only integer values, among which 5 contain a −1, and it is a single −1 in all cases (none of these are optimal in the sense of attaining the lower bound *β*). The other 523 are binary (have all entries in {0,1}), and of those, 34 are not permutation matrices; as expected from Corollary 1 they all contain a single 1 per row, but they each contain multiple 1’s in 1 or 2 columns (and none of these are optimal). Of the final 489, 3 are permutation matrices that are not involutions (i.e. genomic matrices), and interestingly, all of these are optimal and are only found by our algorithm, not the one in [23]. The final 486 are genomic matrices, and are optimal in all except 7 cases; in those 7 cases, both our algorithm and the one in [23] are off by 1 from the optimal bound *β*. The final 479 outputs are both genomic and optimal.

Third, we observe that our compressed sensing-based algorithm produces strictly more optimal solutions than the one in [23], namely, 493 instead of 473 - this is reassuring as it shows that our compressed sensing algorithm can also be optimal in cases where the original one fails (the other way around is not possible due to Theorem 1). However, in the non-optimal cases, the approximation ratio of the original algorithm tends to be lower; this occurs in 39 cases, and 8 other cases result in ties. This is described in Table 2.

**Table 2:** Average percentage excess over the lower bound *β*; the first number denotes our algorithm, while the second one (in brackets) represents the algorithm by Pereira Zanetti *et al* [23]

*n,r*	0.05	0.1	0.15	0.2	0.25	0.3
12	0 (0)	3.3 (1.7)	0 (0)	2 (2.9)	3.64 (1.8)	9.3 (5.1)
16	0 (0)	0 (0)	0 (0)	0 (0)	3 (2.3)	0.7 (1.4)
20	0 (0)	0 (0)	0 (0)	2.3 (1.5)	2.1 (2.8)	6.4 (2.9)
30	3.3 (1.1)	2.2 (1.1)	2 (0.7)	1.9 (1)	1.1 (0.9)	3.7 (2.3)
50	0 (0)	0 (0)	1.1 (0.4)	0.9 (0.3)	1.6 (1)	1.8 (1.5)
100	0 (0)	0.8 (0.3)	0 (0)	0.8 (0.4)	0 (0.2)	0.8 (0.3)
200	0 (0)	0 (0)	0 (0)	0.3 (0.2)	0.1 (0.2)	0.2 (0.3)
300	0 (0)	0 (0)	0.1 (0.1)	0 (0.1)	0.4 (0.2)	0.1 (0.1)
500	0 (0)	0 (0)	0.1 (0.1)	0 (0)	0 (0.1)	0.1 (0.1)

As can be seen from this table, both algorithms tend to be very close to the optimal, but there is no consistent winner between them; therefore, it might make sense to pick the better-scoring candidate among their respective outputs when the best median candidate is desired. Alternatively, when more time is available for the computation, the exact median-finding algorithm presented in this paper should be used instead.

As expected from the proofs in Additional file 1, the exact *O*(*n*^4^) algorithm always finds the median that achieves the lower bound *β*. This algorithm tends to run quickly for small problem sizes (up to *n*=100), averaging less than 0.1 s per instance, about 3 s for *n*=200, 13 s for *n*=300, and 96 s for *n*=500.

For comparison, we also ran the simulated dataset through an exact DCJ median solver, ASMedian-linear [47]. This software tends to consume a large amount of resources, especially for larger instances, or for instances far from each other. As a result, we had to limit its resource consumption to 100 GB of disk space per instance. With this restriction, 86 instances failed to finish, distributed as follows among the input sizes: 8 of size 100, 23 of size 200, 26 of size 300, and 29 of size 500. Considering only the 454 instances that did complete the job, the average time per instance in each size range was as follows: 0.2 seconds for small sizes (up to *n*=50, where all instances finished without an issue), 34 s for *n*=100, 43 s for *n*=200, 12 s for *n*=300, and 60 s for *n*=500. The average time per instance seems to be comparable to the exact algorithm, but our sampling of instances that completed the job is biased. Instances that did not finish typically ran for about 20 min before running out of space. The results are shown in Table 3.

**Table 3:** Average difference and ratio between scores

Size	Instances	D_*ex*_−D_*AS*_	D_*ex*_/D_*AS*_	R_*AS*_−R_*ex*_	R_*AS*_/R_*ex*_
12–50	117	0.21	1.03	0.47	1.04
100	10	0	1	0	1
200	8	0	1	0	1
300	10	0	1	0.1	1
500	6	0	1	0	1
All sizes	151	0.17	1.02	0.37	1.04

We analyze the 151 instances where the exact algorithm produced genomes and ASMedian-linear executed within the resource bounds. D _*ex*_ (resp. R _*ex*_) is the DCJ score (resp. rank score) of the solution generated by the exact algorithm. D _*AS*_ (resp. R _*AS*_) is the DCJ score (resp. rank score) of the solution generated by ASMedian-linear. Each entry shows the average value for all instances of the corresponding size (first column). The number of instances in each size range are shown in the “Instances” column. Notice that all scores are very close. The average difference never surpasses 0.5, and the average ratio is always within 4% of 1. Since the exact algorithm produces optimum rank scores and ASMedian-linear produces optimum DCJ scores, we always use the smaller score as reference. Therefore, all differences are non-negative and all ratios are greater than or equal to 1

### Real dataset

The real dataset consists of a set of 12 Campanulaceæ chloroplast genomes as well as the Tobacco chloroplast genome. We create all possible triples of inputs from this dataset, for a total of 286 input samples; each input had 105 genes, or *n*=210 extremities.

The total time required for processing all the samples with the compressed sensing algorithm was 75 s, or less than 0.3 s per sample on average, which is consistent with the running times we obtained on simulated data.

Among the 286 test cases, 103 had some fractional output values. A total of 2448 entries among them, or 0.05% of the total, were fractions, and they included $\pm \frac {1}{2}, \pm \frac {1}{4}, \pm \frac {1}{5}, \frac {2}{5}, \frac {3}{5}$ and $\frac {3}{4}$. Just over half of them, 52 out of 103, had a score that attained the lower bound *β*.

Of the remaining 183 median candidates, 3 had a single −1 value in the output and were not optimal. Another 15 were binary but not permutation matrices (most with multiple 1s in 1 or 2 columns, and one occurrence in which there were multiple 1s in 3, 4 and 5 columns, respectively), and those were also not optimal. The remaining 165 were genomic matrices (there were no non-genomic permutation matrices), and all of these were optimal.

On real data, our compressed sensing algorithm again outperformed the one in [23] in terms of the number of optimal medians (those with score *β*) found - 217 vs. 189 out of 286; however, it did not perform as well in terms of the average ratio between the obtained score and the lower bound *β* - the average was 3% above *β* for the compressed sensing algorithm vs. 2% above *β* for the original algorithm. Our algorithm had a higher score more often, 57 vs. 32 out of 286 times, with the remaining 197 being ties. Once again, the choice of algorithm depends on the user’s preference for a higher chance of getting an exact median vs. a better approximation ratio, and the optimal method seems to be to pick the best-scoring output among the two algorithms. If time is not of the essence or the input problem size is relatively small, then the exact median-finding algorithm should be used.

The total time required for processing all the samples with the exact polynomial-time algorithm was 85 seconds, or less than 0.3 seconds per sample on average, which is extremely fast. All the computations converged without numerical stability issues, and required between 0 and 16 iterations, with an average of 6 iterations. With this algorithm, 106 out of 286 cases had some fractional output values, while 170 were genomic and 10 others were non-genomic permutation matrices.

Among the 106 results with fractional output values, a total of 4700 entries, or 0.1% of the total, were non-integers. Some patterns emerged for which fractions were present - the most frequently found fractional entries were $\pm \frac {1}{2}$, while others included $\pm \frac {1}{8},\pm \frac {1}{7}, \pm \frac {1}{6}, \pm \frac {1}{4}, \pm \frac {2}{7}, \pm \frac {1}{3}, \pm \frac {3}{8}, \pm \frac {3}{7}, \pm \frac {4}{7}, \frac {5}{8}, \frac {2}{3}, \frac {5}{7}, \frac {3}{4}, \frac {5}{6}$. However, there were some sporadic fractional values with larger denominators such as 10, 12, 14, 17, and 20, as well as some irrational numbers likely introduced by the normalization of an orthogonal basis during the computation. All the computed medians attained the lower bound, as expected from the theoretical results described above.

## Discussion

### When the median is not genomic

The fact that matrix medians of genome inputs are not always genomic is surprising, but we offer here an interpretation of this result that can be helpful. Even when it is not a genome, the output of our exact algorithm is always orthogonal. It follows that, in each row, the squares of the entries are nonnegative numbers that add to 1. They can therefore be seen as a *probability distribution* on the potential adjacencies. We can use this fact to sample adjacencies row by row, avoiding common extremities, and construct a genome.

For instance, consider the genomes

$${} A = \left[\begin{array}{cccc} 0 & 1 & 0 & 0 \\ 1 & 0 & 0 & 0 \\ 0 & 0 & 0 & 1 \\ 0 & 0 & 1 & 0 \end{array}\right], B = \left[\begin{array}{cccc} 0 & 0 & 0 & 1 \\ 0 & 0 & 1 & 0 \\ 0 & 1 & 0 & 0 \\ 1 & 0 & 0 & 0 \end{array}\right], C = \left[\begin{array}{cccc} 0 & 0 & 1 & 0 \\ 0 & 0 & 0 & 1 \\ 1 & 0 & 0 & 0 \\ 0 & 1 & 0 & 0 \end{array}\right]. $$

The unique median of these three matrices is the following matrix, which will be returned by the exact algorithm:

$$M = \left[\begin{array}{cccc} -0.5 & 0.5 & 0.5 & 0.5 \\ 0.5 & -0.5 & 0.5 & 0.5 \\ 0.5 & 0.5 & -0.5 & 0.5 \\ 0.5 & 0.5 & 0.5 & -0.5 \end{array}\right]. $$

Squaring each entry, we get (∘ denotes the component-wise product)

$$M \circ M = \left[\begin{array}{cccc} 0.25 & 0.25 & 0.25 & 0.25 \\ 0.25 & 0.25 & 0.25 & 0.25 \\ 0.25 & 0.25 & 0.25 & 0.25 \\ 0.25 & 0.25 & 0.25 & 0.25 \end{array}\right]. $$

If we sample from this distribution, we can choose any set of adjacencies (provided they have distinct extremities), since they are all equally likely. If we choose all adjacencies outside the main diagonal, we end up with either *A*, *B*, or *C*. They all have score 4, the best among genomes. So, we reach a genomic median in this case. If we choose exactly two adjacencies from the diagonal, we end up with a genome with score 7. If we choose all of them on the diagonal, we end up with the identity matrix *I*, which has score 6. It seems that a good strategy then is to use the probabilities of *M*∘*M* to sample, but also to use *M* to avoid its negative entries. Exploring this idea further is beyond the scope of this paper, but could be a good topic for further research.

## Conclusion

In this paper we introduced a new algorithm for the median-of-three problem relative to the rank distance based on a necessary condition for attaining the lower bound, and used it to prove the uniqueness of the median in a favorable regime. In addition, we introduced the first polynomial-time algorithm for finding an exact median of three genomes (or three permutations) with respect to the rank distance, placing it in the rare class of tractable rearrangement distances. While the median of three genomes with respect to the rank distance is not always genomic, it intriguingly appears to be a lot of the time.

There are several remaining open questions, which we list here.

- What is the approximation ratio of the compressed sensing algorithm here?
- Are there optimality conditions less restrictive than the one we found?
- What is the best way to convert a non-genomic median matrix to a genome?
- What is the complexity of finding the genomic median of 3 genomic matrices?

## Additional files

Additional file 1 Proofs of results. This Additional File, in PDF format, contains proofs of the following results: Lemma 1, Correctness of the exact algorithm. (PDF 156 kb)

Additional file 2Example of numerical unstability of “triple-null” formula in the Implementation section. This Additional file, in text format suitable to be read by Octave or MATLAB, contains three 100×100 matrices *A*,*B*, and *C* for which the triple-null formula does not work, and the alternative single-null formula for the intersection $\text {im}(A-B) \cap \text {im}(C-B)$ was used. The file can be checked in Octave as follows: Save it as triple.mat, Call Octave, and then type in its prompt: > load(“triple.mat”)
> meet = null([(null(A’-B’))’;(null(C’-B’))’]);

> size(meet)

ans =

100 0

> meet = orth((A-B)*null([A-B C-B])(1:100,:));

> size(meet)

ans =

100 2
This shows that, while the first formula produced a zero-dimensional subspace, the second one actually produced a 2-dimensional subspace. (MAT 222 kb)
