# Supplementary material for: On the rank-distance median of 3 permutations
Source: BMC Bioinformatics. 2018 May 8;19(Suppl 6):142. doi: 10.1186/s12859-018-2131-4 (PMC5998913; doi:10.1186/s12859-018-2131-4)
Supplement: Supplementary file 1 — Proofs of results. This Additional File, in PDF format, contains proofs of the following results: Lemma 1, Correctness of the exact algorithm. (PDF 156 kb) [file 12859_2018_2131_MOESM1_ESM.pdf]

Additional File 1 for "On the Rank-Distance Median of 3 Permutations"

Leonid Chindelevitch João Paulo Pereira Zanetti
João Meidanis

Proof of Lemma 1

Proof. Clearly, $r(X) \geq 0 \; \forall \; X$, and $r(A-B) = 0 \iff A-B = 0 \iff A = B$.
The symmetry follows from $d(A,B) = r(A-B) = r(B-A) = d(B,A)$.
The triangle inequality follows from the fact that the rank of X is the vector space dimension of its *image* $\text{im}(X) := \{Xv | v \in \mathbb{R}^n\}$, so it suffices to show that

$$\dim(\text{im}(X)) + \dim(\text{im}(Y)) \geq \dim(\text{im}(X+Y))$$

and substitute $X = B - A$ and $Y = C - B$ to conclude that

$$d(A,B) + d(B,C) \geq d(A,C) \; \forall \; A, B, C \in \mathbb{R}^{n \times n}.$$

Now, it is easy to see that

$$\begin{aligned} \text{im}(X+Y) &= \{(X+Y)v | v \in \mathbb{R}^n\} = \{(Xv + Yv | v \in \mathbb{R}^n\} \\ &\subseteq \{Xv | v \in \mathbb{R}^n\} + \{Yv | v \in \mathbb{R}^n\} = \text{im}(X) + \text{im}(Y), \end{aligned}$$

where the addition in the second line is the addition of vector spaces. Therefore,

$$\begin{aligned} r(X+Y) &= \dim(\text{im}(X+Y)) \\ &\leq \dim(\text{im}(X) + \text{im}(Y)) \\ &= \dim(\text{im}(X)) + \dim(\text{im}(Y)) - \dim(\text{im}(X) \cap \text{im}(Y)) \\ &\leq \dim(\text{im}(X)) + \dim(\text{im}(Y)) \\ &= r(X) + r(Y), \end{aligned}$$

where the equality follows from properties of vector space addition [1]. In particular, equality happens if and only if both of the following conditions hold:

$$\dim(\operatorname{im}(X+Y)) = \dim(\operatorname{im}(X)+\operatorname{im}(Y)) \iff \operatorname{im}(X+Y) = \operatorname{im}(X)+\operatorname{im}(Y);$$
$$\dim(\operatorname{im}(X)\cap\operatorname{im}(Y)) = 0 \iff \operatorname{im}(X)\cap\operatorname{im}(Y) = \{0\}.$$

These two conditions are equivalent to the vector space $\operatorname{im}(X+Y)$ being the *direct sum* of $\operatorname{im}(X)$ and $\operatorname{im}(Y)$, written as $\operatorname{im}(X+Y) = \operatorname{im}(X) \oplus \operatorname{im}(Y)$.

To finish the proof, let us pick a basis $\mathbb{B}_X$ for $\operatorname{im}(X)$ and a basis $\mathbb{B}_Y$ for $\operatorname{im}(Y)$. From the directness of the sum, it follows that every vector $u \in \operatorname{im}(X+Y)$ can be uniquely written as $u = v + w$, with $v \in \operatorname{im}(X)$ and $w \in \operatorname{im}(Y)$. Since this is true for any vector in $\operatorname{im}(X+Y)$, this is in particular true for the n columns of $X+Y$, say $c_1, \ldots, c_n$.

But we also have

$$c_i = x_i + y_i \; \forall \; 1 \leq i \leq n, \tag{1}$$

where x_i (respectively y_i) is the i-th column of X (respectively Y). Therefore, since $x_i \in \operatorname{im}(X)$ and $y_i \in \operatorname{im}(Y)$, Equation (1) gives the desired decomposition for each c_i. Let $\mathbb{B} := \mathbb{B}_X \cup \mathbb{B}_Y$ be the basis of $\operatorname{im}(X+Y)$ obtained by joining the bases $\mathbb{B}_X$ and $\mathbb{B}_Y$, and let $\mathbb{B}^+ := \mathbb{B} \cup \mathbb{B}'$ be an extension of the basis $\mathbb{B}$ to a basis of the entire space $\mathbb{R}^n$. Let P be the matrix of the projection operator onto X with respect to the basis $\mathbb{B}^+$, with all the elements in $\mathbb{B}' = \mathbb{B}^+ - \mathbb{B}$ being projected onto the 0 vector. Then

$$Pc_i = x_i \; \forall \; 1 \leq i \leq n,$$

so that $P(X+Y) = X$. By substituting $X = B - A$ and $Y = C - B$, we see that the desired equality is only possible if $B = A + P(C-A)$, as required. □

Proofs related to the exact $O(n^4)$ algorithm

The remaining proofs in this Additional File concern the exact, $O(n^4)$ algorithm.

The subspace $\operatorname{im}(A-B) \cap \operatorname{im}(C-B)$

An important observation here is that Lemmas 4-6 and Theorem 2 from Pereira Zanetti et al. [2], restated in Equation 6 of the “On the Rank-Distance Median

of 3 Permutations" paper, and originally proven for permutation matrices, are also valid for orthogonal matrices, with the exact same proof. The fundamental fact that is the basis for all these proofs is the fact that permutation matrices preserve norms, something that is also true for orthogonal matrices.

Theorem 1. *If A, B, and C are three $n \times n$ orthogonal matrices, then*

$$\dim(\operatorname{im}(A-B) \cap \operatorname{im}(C-B)) \geq (d(A,B) + d(B,C) - d(A,C))/2.$$

Proof.

$$\begin{aligned}
\dim(\operatorname{im}(A-B) \cap \operatorname{im}(C-B)) &= n - \dim((\operatorname{im}(A-B) \cap \operatorname{im}(C-B))^{\perp}) \\
&= n - \dim(\operatorname{im}(A-B)^{\perp} + \operatorname{im}(C-B)^{\perp}) \\
&= n - \dim(\operatorname{im}(A-B)^{\perp}) - \dim(\operatorname{im}(C-B)^{\perp}) \\
&\quad + \dim(\operatorname{im}(A-B)^{\perp} \cap \operatorname{im}(C-B)^{\perp}) \\
&= n - (n - d(A,B)) - (n - d(C,B)) \\
&\quad + \dim(\ker(A-B) \cap \ker(C-B)) \\
&= d(A,B) + d(B,C) - n + \alpha \\
&\geq d(A,B) + d(B,C) - \beta \\
&= (d(A,B) + d(B,C) - d(A,C))/2.
\end{aligned}$$

□

The above derivation uses the formulas for the dimension of $V^{\perp}$ given the dimension of V, the fact that $(V_1 + V_2)^{\perp} = V_2^{\perp} \cap V_2^{\perp}$, the definition of rank distance, Lemma 1 below, the fact that $(\operatorname{im} X)^{\perp} = \ker(X^T)$, the definitions of invariants α and β, and the fact that $\alpha + \beta \geq n$.

Lemma 1. *If A, B, and C are three $n \times n$ orthogonal matrices, then*

$$\ker(A^T - B^T) \cap \ker(C^T - B^T) = B(\ker(A-B) \cap \ker(C-B)).$$

Proof. If $x \in \ker(A^T - B^T) \cap \ker(C^T - B^T)$ we have $A^T x = B^T x = C^T x$. Then $BB^T x = x = AA^T x = AB^T x$, showing that $B^T x \in \ker(A-B)$. Similarly, $BB^T x = x = CC^T x = CB^T x$, showing that $B^T x \in \ker(C-B)$ as well. Therefore $x = BB^T x \in B(\ker(A-B) \cap \ker(C-B))$.

Conversely, if $x \in \ker(A-B) \cap \ker(C-B)$ we have $Ax = Bx = Cx$. Then $B^T Bx = x = A^T Ax = A^T Bx$, showing that $Bx \in \ker(A^T - B^T)$. Similarly, $B^T Bx = x = C^T Cx = C^T Bx$, showing that $Bx \in \ker(C^T - B^T)$ as well. It follows that $Bx \in \ker(A^T - B^T) \cap \ker(C^T - B^T))$. □

Decomposition of $A - B$ and $C - B$

In this section we study the decomposition of a generic $n \times n$ matrix into a minimal sum of rank 1 matrices. This is important for our problem because if we want to take a step towards A from B, then looking at optimal rank-1 decompositions of $A - B$ will help.

If H and F are two $n \times n$ matrices, we say that H **decomposes** F when $r(H) = 1$ and $r(F - H) < r(F)$. This means that H is one possible term in a decomposition

$$F = u_1 w_1^T + u_2 w_2^T + \ldots + u_k w_k^T,$$

where $k = r(F)$.

We begin with a characterization of all such possible terms.

Theorem 2. *A matrix H decomposes F if and only if $H = Fxy^T F$, where x and y are vectors such that $y^T Fx = 1$.*

Proof. ($\Longrightarrow$)

Notice that $F - H = F(I - xy^T F)$, so $\mathrm{im}(F - H) \subseteq \mathrm{im}\, F$ and therefore $r(F - H) \leq r(F)$.

On the other hand, $Fx \in \mathrm{im}\, F$ and $Fx \notin \mathrm{im}(F - H)$, because $Fx = (F - H)z$ leads to a contradiction if we multiply by y^T on the left. So, in fact, $r(F - H) < r(F)$.

($\Longleftarrow$)

If H decomposes F we can write

$$F = u_1 w_1^T + u_2 w_2^T + u_3 w_3^T + \ldots + u_k w_k^T, \tag{2}$$

where $H = u_1 w_1^T$ and $k = r(F)$. By applying F to a suitable vector x orthogonal to $w_2, w_3, \ldots, w_k$ and such that $w_1^t x = 1$, we end up with

$$Fx = u_1.$$

By multiplying Equation (2) on the left by y^T for a suitable vector y orthogonal to $u_2, u_3, \ldots, u_k$ and such that $y^T u_1 = 1$, we end up with

$$y^T F = w_1^T.$$

Hence, $H = u_1 w_1^T = Fxy^T F$, and $y^T Fx = y^T u_1 = 1$. □

Let us now apply this result to the case in hand. Suppose $u \in \mathrm{im}(A - B) \cap \mathrm{im}(C - B)$ is different from zero. We claim that $H = -2uu^T B / u^T u$ decomposes $A - B$ and also $C - B$.

Theorem 3. *If A, B, and C are three $n \times n$ orthogonal matrices and $u \in \operatorname{im}(A-B) \cap \operatorname{im}(C-B)$ is different from zero, then $H = -2uu^T B / u^T u$ decomposes both $A - B$ and $C - B$.*

Proof. Since the expression for H is invariant under multiplication of u by a nonzero scalar, we may, without loss of generality, assume u is unitary, that is, $u^T u = 1$.

Let us first show that H decomposes $A - B$. Given that $u \in \operatorname{im}(A - B)$, there must be a vector x such that $u = (A - B)x$. Take $y = 2Ax$. We claim that $y^T (A - B)x = 1$. This can be verified by expanding both $y^T u$ and $u^T u$, which end up being the same thing. Hence, $y^T (A - B)x = y^T u = u^T u = 1$.

With this, we are ready to apply Theorem 2 for $F = A-B$, to conclude that $H = (A-B)xy^T(A-B)$ decomposes $A-B$. However, $(A-B)x = u$ by definition and $y^T(A-B) = -2u^T B$ can be verified by expanding both expressions, which end up being equal to $2x^T - 2x^T A^T B$. Then $H = (A - B)xy^T(A - B) = -2uu^T B$, as claimed.

Finally, notice that, after the choice of u, matrix H depends only on u and B, and therefore the conclusion applies to $C - B$ as well. □

Walking towards a median

In this section we will prove the following result.

Theorem 4. *If A, B, and C are three $n \times n$ matrices, and B_1 is a matrix of the same size such that*

$$d(A, B_1) + d(B_1, B) = d(A, B)$$

and

$$d(C, B_1) + d(B_1, B) = d(C, B)$$

then any median of A, B_1, and C that satisfies the lower bound $\beta(A, B_1, C)$ is also a median of A, B, and C that satisfies their lower bound $\beta(A, B, C)$.

Proof. Let M be a median of A, B_1, and C that satisfies the lower bound, namely

$$d(A, M) + d(B_1, M), +d(C, M) = \frac{1}{2}(d(A, B_1) + d(B_1, C), +d(C, A)).$$

We know that this entails

$$\begin{aligned} d(A, M) + d(M, B_1) &= d(A, B_1), \\ d(B_1, M) + d(M, C) &= d(B_1, C), \\ d(C, M) + d(M, A) &= d(C, A). \end{aligned}$$

But then

$$\begin{aligned} d(A, M) + d(M, B) &= d(A, B_1) - d(M, B_1) + d(M, B) \\ &= d(A, B) - d(B, B_1) - d(M, B_1) + d(M, B) \\ &\leq d(A, B). \end{aligned}$$

Similarly, $d(B, M) + d(M, C) \leq d(B, C)$. We conclude that M satisfies also the lower bound for A, B, and C and is therefore a median of these three matrices. □

Correctness proof

Here we wrap up the previous results in the main correctness proof. Before embarking on this proof, we need the generalization to orthogonal matrices of Lemma 3 of the "On the Rank-Distance Median of 3 Permutations" paper, which states the integrality of the invariant β. This can be proven as follows. It is known that every orthogonal matrix T is the product of Householder transformations. We strengthen this result to show that we can always use exactly k factors in this product, where $k = \dim \operatorname{im}(T - I)$, in Theorem 5 below. Since each Householder transformation has determinant -1, it follows that $\det(T) = (-1)^{d(T,I)}$, or, for arbitrary orthogonal matrices A and B, $\det(A^{-1}B) = (-1)^{d(A,B)}$. With this result, the alternative proof of the integrality of β provided in the aforementioned Lemma 3 extends to orthogonal matrices.

Definition 1. *Let $v \neq 0$ be a vector of $\mathbb{F}^n$, where $\mathbb{F}$ is a field of characteristic zero. The* ***Householder transformation*** *along v is defined as the matrix*

$$H(v) = I - 2vv^T / v^T v.$$

Properties:

- $H(v)^T = H(v)$

- $d(H(v), I) = 1$
- $H(v)^2 = I$

These properties are all well-known and easy to prove from the definitions, with straightforward calculations. Householder transformations are in fact reflections with respect to the hyperplane orthogonal to v. As such, they have determinant equal to -1, because they can be diagonalized with one eigenvector relative to eigenvalue -1 (in the direction of v), and $n-1$ eigenvectors relative to eigenvalue $+1$ (in the hyperplane orthogonal to v). Now comes an important theorem related to the factoring of an orthogonal matrix as a product of such hyperplane reflections.

Lemma 2. *If $T \neq I$ is an orthogonal matrix, there exists a Householder transformation H such that $d(HT, I) < d(T, I)$.*

Proof. We sketch the proof. Take any unitary vector u in $\ker(T-I)^{\perp}$, and define $H = H(v)$, where $v = Tu - u$. Now, if $w \in \ker(T-I)$, then $Tw = w$ and we can prove that $v^T w = 0$, and therefore $Hw = w$ and $HTw = Hw$. We conclude that $\ker(T-I) \subseteq \ker(HT-I)$. The next step is to prove that vector u belongs to the latter but not to the former, leading to

$$\dim\ker(T-I) < \dim\ker(HT-I).$$

But this in turn implies that $d(HT, I) = \dim \operatorname{im}(HT-I) < \dim \operatorname{im}(T-I) = d(T, I)$, which is what we wanted to prove.

As for showing that $u \in \ker(HT-I)$, notice that $Hu = Tu$, since:

$$Hu = (I - 2vv^T/v^T v)u = u - 2vv^T u/v^T v = u - (u - Tu) = Tu.$$

This is because $2vv^T u = (2 - 2u^T Tu)(u - Tu)$ and $v^T v = (2 - 2u^T Tu)$. But if $Hu = Tu$, then $HTu = u$. □

Theorem 5. *Any orthogonal matrix T can be written as a product of k Householder transformations, where $k = d(T, I)$.*

Proof. We show this by induction on k. If $k = 0$, then $T = I$ is indeed the product of zero Householder transformations. If $k > 1$, then there is at least one unitary vector in $\ker(T-I)^{\perp}$, and therefore Theorem 2 tells us that there is a Householder transformation H such that $d(HT, I) < d(T, I)$. Notice that $d(HT, I)$ cannot be smaller than $d(T, I) - 1$, because of the triangle inequality:

$$d(T, I) \leq d(T, HT) + d(HT, I) = 1 + d(HT, I),$$

since $d(T, HT) = d(I, H) = 1$ due to the fact that T is invertible. It follows that $d(HT, I) = k - 1$ and we can apply the induction hypothesis to HT, which is orthogonal because both H and T are, obtaining $k - 1$ Householder transformations H_1, H_2, ..., H_{k-1} such that

$$HT = H_1 H_2 \dots H_{k-1}.$$

Since $H^2 = I$, we conclude that $T = HH_1H_2 \dots H_{k-1}$ is a product of k Householder transformations. □

Now our main result.

Theorem 6. *The matrix M returned by Algorithm 1 of the "On the Rank-Distance Median of 3 Permutations" paper is orthogonal and satisfies the lower bound for A, B, and C.*

Proof. By induction on $k = d(A, B) + d(B, C) - d(C, A)$. Notice that this number is nonnegative by the triangle inequality. If $k = 0$, the algorithm returns B, which is orthogonal and satisfies the lower bound, since it lies on a shortest path between A and C. If $k = 1$ the conclusion is vacuously true since this case is impossible (k is always an even number by the integrality of invariant β).

If $k \geq 2$, the algorithm finds a non-zero vector u in $\mathrm{im}(A - B) \cap \mathrm{im}(C - B)$, which exists because of Theorem 1. It then computes $H = -2uu^T B/u^T u$ and $B+H$, which stands on a shortest path between B and A, and also on a shortest path between B and C, according to Theorem 3.

The matrix $B + H$ is orthogonal, as we can readily verify:

$$(B + H)(B + H)^T = \left(I - \frac{2uu^T}{u^T u}\right) BB^T \left(I - \frac{2uu^T}{u^T u}\right)^T = \left(I - \frac{2uu^T}{u^T u}\right)^2 = I,$$

where we used the symmetry and idempotency of Householder reflections.

Therefore $B+H$ is indeed orthogonal and the recursive call is valid, because all arguments are orthogonal matrices. But since

$$d(A, B+H)+d(B+H, C)-d(A, C) = d(A, B)-1+d(B, C)-1-d(A, C) = k-2,$$

we can apply the induction hypothesis and conclude that the median M returned for A, $B + H$, and C is orthogonal and satisfies the lower bound for these matrices. Using Theorem 4, we conclude that M also satisfies the lower bound for A, B, and C, and is therefore a median. Since M is orthogonal by the induction hypothesis, we are done. □

References

[1] Roman, S.: Advanced Linear Algebra. Graduate Texts in Mathematics. Springer, New York, NY (2008)

[2] Zanetti, J.P.P., Biller, P., Meidanis, J.: Median approximations for genomes modeled as matrices. Bulletin of Math Biology **78**(4) (2016)
